# Genome-wide meta-analysis of ascertainment and symptom structures of major depression in case-enriched and community cohorts

**DOI:** 10.1017/S0033291724001880

**Published:** 2024-09

**Authors:** Mark J. Adams, Jackson G. Thorp, Bradley S. Jermy, Alex S. F. Kwong, Kadri Kõiv, Andrew D. Grotzinger, Michel G. Nivard, Sally Marshall, Yuri Milaneschi, Bernhard T. Baune, Bertram Müller-Myhsok, Brenda W. J. H. Penninx, Dorret I. Boomsma, Douglas F. Levinson, Gerome Breen, Giorgio Pistis, Hans J. Grabe, Henning Tiemeier, Klaus Berger, Marcella Rietschel, Patrik K. Magnusson, Rudolf Uher, Steven P. Hamilton, Susanne Lucae, Kelli Lehto, Qingqin S. Li, Enda M. Byrne, Ian B. Hickie, Nicholas G. Martin, Sarah E Medland, Naomi R. Wray, Elliot M. Tucker-Drob, Estonian Biobank Research Team, Major Depressive Disorder Working Group of the Psychiatric Genomics Consortium, Cathryn M. Lewis, Andrew M McIntosh, Eske M. Derks

**Affiliations:** 1 Division of Psychiatry, University of Edinburgh, Edinburgh, UK; 2 Mental Health and Neuroscience, QIMR Berghofer Medical Research Institute, Brisbane, QLD, Australia; 3 Institute for Molecular Medicine Finland, University of Helsinki, Helsinki, Finland; 4 MRC Integrative Epidemiology Unit, University of Bristol, Bristol, UK; 5 Estonian Genome Centre, Institute of Genomics, University of Tartu, Tartu, Estonia; 6 Department of Psychology and Neuroscience, University of Colorado at Boulder, Boulder, CO, USA; 7 Institute for Behavioral Genetics, University of Colorado at Boulder, Boulder, CO, USA; 8 Department of Biological Psychology, Vrije Universiteit Amsterdam, Amsterdam, Netherlands; 9 Centre for Genomic & Experimental Medicine, Institute of Genetics and Cancer, University of Edinburgh, Edinburgh, UK; 10 Department of Psychiatry, Amsterdam Public Health and Amsterdam Neuroscience, Amsterdam UMC, Vrije Universiteit Amsterdam, Amsterdam, Netherlands; 11 Department of Psychiatry, University of Melbourne, Melbourne, VIC, Australia; 12 Florey Institute of Neuroscience and Mental Health, University of Melbourne, Melbourne, VIC, Australia; 13 Department of Psychiatry, University of Münster, Münster, NRW, Germany; 14 Department of Translational Research in Psychiatry, Max Planck Institute of Psychiatry, Munich, BY, Germany; 15 Munich Cluster for Systems Neurology (SyNergy), Munich, BY, Germany; 16 Institute of Population Health, University of Liverpool, Liverpool, UK; 17 Department of Biological Psychology & Amsterdam Public Health Research Institute, Vrije Universiteit Amsterdam, Amsterdam, Netherlands; 18 Department of Psychiatry & Behavioral Sciences, Stanford University, Stanford, CA, USA; 19 Social, Genetic and Developmental Psychiatry Centre, King's College London, London, UK; 20 NIHR Maudsley Biomedical Research Centre, King's College London, London, UK; 21 Department of Psychiatry, Lausanne University Hospital and University of Lausanne, Prilly, VD, Switzerland; 22 Department of Psychiatry and Psychotherapy, University Medicine Greifswald, Greifswald, MV, Germany; 23 Child and Adolescent Psychiatry, Erasmus University Medical Center Rotterdam, Rotterdam, Netherlands; 24 Social and Behavioral Science, Harvard T.H. Chan School of Public Health, Boston, MA, USA; 25 Institute of Epidemiology and Social Medicine, University of Münster, Münster, NRW, Germany; 26 Department of Genetic Epidemiology in Psychiatry, Central Institute of Mental Health, Medical Faculty Mannheim, Heidelberg University, Mannheim, BW, Germany; 27 Department of Medical Epidemiology and Biostatistics, Karolinska Institutet, Stockholm, Sweden; 28 Psychiatry, Dalhousie University, Halifax, NS, Canada; 29 Psychiatry, Kaiser Permanente Northern California, San Francisco, CA, USA; 30 Max Planck Institute of Psychiatry, Munich, BY, Germany; 31 Neuroscience Therapeutic Area, Janssen Research and Development, LLC, Titusville, NJ, USA; 32 Child Health Research Centre, University of Queensland, Brisbane, QLD, Australia; 33 Brain and Mind Centre, University of Sydney, Sydney, NSW, Australia; 34 Institute for Molecular Bioscience, University of Queensland, Brisbane, QLD, Australia; 35 Queensland Brain Institute, University of Queensland, Brisbane, QLD, Australia; 36 Department of Psychology, University of Texas at Austin, Austin, TX, USA; 37 Population Research Center, University of Texas at Austin, Austin, TX, USA;; 38 Department of Medical & Molecular Genetics, King's College London, London, UK; 39 Institute for Genomics and Cancer, University of Edinburgh, Edinburgh, UK

**Keywords:** depressive symptoms, genome-wide association study, Genomic SEM, major depressive disorder, psychometrics

## Abstract

**Background:**

Diagnostic criteria for major depressive disorder allow for heterogeneous symptom profiles but genetic analysis of major depressive symptoms has the potential to identify clinical and etiological subtypes. There are several challenges to integrating symptom data from genetically informative cohorts, such as sample size differences between clinical and community cohorts and various patterns of missing data.

**Methods:**

We conducted genome-wide association studies of major depressive symptoms in three cohorts that were enriched for participants with a diagnosis of depression (Psychiatric Genomics Consortium, Australian Genetics of Depression Study, Generation Scotland) and three community cohorts who were not recruited on the basis of diagnosis (Avon Longitudinal Study of Parents and Children, Estonian Biobank, and UK Biobank). We fit a series of confirmatory factor models with factors that accounted for how symptom data was sampled and then compared alternative models with different symptom factors.

**Results:**

The best fitting model had a distinct factor for *Appetite/Weight* symptoms and an additional measurement factor that accounted for the skip-structure in community cohorts (use of Depression and Anhedonia as gating symptoms).

**Conclusion:**

The results show the importance of assessing the directionality of symptoms (such as hypersomnia versus insomnia) and of accounting for study and measurement design when meta-analyzing genetic association data.

## Introduction

Major depressive disorder (MDD) is a mood disorder characterized by low mood, loss of interest or pleasure (anhedonia), irritable affect, biological symptoms (psychomotor agitation/slowing, altered sleep patterns, changes in appetite or weight), negative thought content, and associated loss of function. To qualify for a diagnosis of major depression, the standard diagnostic classification systems (American Psychiatric Association, 2000, 2013; World Health Organization, 1992) require one of two cardinal symptoms plus at least four other symptoms to be present. Although conceptualized as a single disorder, the diagnostic criteria for MDD can be met with any combination of these other symptoms, which entails the potential of hundreds or thousands of symptom profiles (Fried & Nesse, 2015a; Zimmerman, Ellison, Young, Chelminski, & Dalrymple, 2015). A single categorical phenotype – that might mask a multitude of separate disorder types – stymies the testing of correlates and treatments. However, heterogeneity within the MDD diagnosis does have an upper bound: only around one quarter of the potential symptom profiles are actually observed (Fried & Nesse, 2015a; Zimmerman et al., 2015).

Analyzing individual symptoms is one way to unwrap the heterogeneity of MDD (Cai, Choi, & Fried, 2020; Fried & Nesse, 2015b). Phenotypic studies have derived and tested factor structures of MDD symptoms (Elhai et al., 2012; Krause, Bombardier, & Carter, 2008; Krause, Reed, & McArdle, 2010) and twin models have been used to separate genetic from environmental sources of symptom covariance (Kendler, Aggen, & Neale, 2013) and identify the low genetic concordance between symptoms assessed inside and outside of a depressive episode (Kendler & Aggen, 2023). These models grouped symptoms together in two or three factors, which broadly contrast psychological *v.* somatic symptoms. Clinical subtypes are also part of diagnostic criteria and these have been used to classify depression profiles that are differentially associated with specific clinical, behavioral, and biological correlates (Milaneschi, Lamers, Berk, & Penninx, 2020; Penninx, Milaneschi, Lamers, & Vogelzangs, 2013). The context of symptom expression is an additional part of heterogeneity. For example, symptoms like sleep changes can have many causes unrelated to depression.

More recently, genetic studies of depressive symptoms have updated the findings from twin models using data from genome-wide association studies (GWAS). A confirmatory factor analysis of genetic covariance estimates obtained from GWAS results on current depressive symptoms showed that a psychological and somatic factor had the best fit to the data (Thorp et al., 2020). The detection of genetic correlates specific to each symptom implies that symptoms may have differing genetic causes and consequences, even if the symptoms themselves are highly genetically correlated.

Understanding the genetic architecture of MDD symptoms is complicated by symptom ascertainment. In clinically ascertained samples, symptom data is often only available on affected participants, and is thus conditioned on having been diagnosed with depression. Conditioning data presence on a diagnosis can induce downward bias in correlations amongst the symptoms comprising that diagnosis, removing any shared genetic component. In community and biobank cohorts, participants are typically screened for the presence of cardinal symptoms (depressed mood and anhedonia) and only participants who report at least one cardinal symptom are assessed for other symptoms of depression, which also leads to high levels of missing symptom data in these cohorts. Because community samples often contain symptom but not diagnostic information, many GWAS purporting to investigate MDD may actually be better characterized as investigating a broader dysphoria continuum rather than MDD specifically (Flint, 2023). However, the use of cardinal symptom screening also potentially enhances the suitability of community cohorts to add to the understanding of non-cardinal symptom dimensions in the context of depression (Huang et al., 2023).

In this study we sought to uncover the genetic structure of depression symptoms while accounting for how samples were recruited and how symptoms were assessed. We did this by conducting GWAS of individual symptoms of depression, testing factor models to investigate genetic heterogeneity as a function of sample ascertainment (Case v. Community cohorts) and measurement (with or without screening based on cardinal/gating symptoms). Finally, we assessed the validity of the identified latent factors of depression by estimating genetic correlations with external traits.

Specifically, we conducted GWAS of symptom data in six cohorts and meta-analyzed them in groups based on sample ascertainment. The first group (the ‘Case-enriched’ cohorts) consisted of clinical cases from the Psychiatric Genomics Consortium MDD cohorts, participants from the Australian Genetics of Depression study who were recruited based on depression diagnosis, and participants from Generation Scotland who met DSM criteria for depression. The second group (the ‘Community’ cohorts) consisted of the Avon Longitudinal Study of Parents and Children, Estonian Biobank, and UK Biobank, and thus contained data on participants who were not recruited with respect to depression status. Using the two sets of meta-analyzed symptom GWASs, we tested factor models that accounted for how the samples were ascertained (Case *v.* Community) and how symptoms were assessed (with or without skip structure based on cardinal symptoms). After understanding the measurements structure of the symptom GWASs, we then compared alternative factor models for the symptoms based on previous literature and diagnostic specifiers for depressive disorders. Using the best fitting overall models, we tested for shared and specific genetic correlates with other psychiatric, behavioral, and metabolic phenotypes that have known genetic links to MDD.

## Methods

### Samples and assessments of depression symptoms

We analyzed depression symptom data in six studies: the Psychiatric Genomics Consortium (PGC) (Major Depressive Disorder Working Group of the Psychiatric GWAS Consortium, 2013; Wray et al., 2018), the Australian Genetics of Depression Study (AGDS) (Byrne et al., 2020; Mitchell et al., 2022), Generation Scotland: Scottish Family Health Study (GS:SFHS) (Smith et al., 2012), the Avon Longitudinal Study of Parents and Children (ASLPAC) (Boyd et al., 2013; Fraser et al., 2013), Estonian Biobank (EstBB) (Leitsalu et al., 2015), and UK Biobank (UKB) (Sudlow et al., 2015). We selected participants from the PGC and GS:SHFS cohorts who met DSM criteria for MDD based on structured diagnostic interviews or clinical assessments of their current or lifetime worst episode. Participants from AGDS were recruited based on history of receiving treatment for depression and were assessed for symptoms during their worst episode using an online questionnaire. The PGC, GS:SHFS, and AGDS samples were enriched for depression cases and were grouped together as ‘Case-enriched’ cohorts. In ALSPAC, current depressive symptoms were prospectively collected by interview in the original children sample. In EstBB and UKB, depression symptoms from worst episode were assessed retrospectively using online surveys. Symptom data in these two cohorts had a skip-structure, where all participants were asked about mood and anhedonia symptoms while only participants who endorsed at least one cardinal symptom were asked about the other DSM symptoms. In addition, in UKB we also used retrospective assessments of prolonged low mood and/or anhedonia from the Touchscreen questionnaire. Data from ALSPAC, EstBB, and UKB samples were included regardless of depression diagnosis and were grouped together as ‘Community cohorts’. Table 1 describes the effective sample size of number of participants with each symptom for each grouping of studies that were analyzed. Effective sample size was calculated within each study as *N*_Eff_ = 4/(1/*N*_Cases_ + 1/*N*_Controls_) and then summed to get total effective sample size for each meta-analysis (Grotzinger, de la Fuente, Privé, Nivard, & Tucker-Drob, 2022). See Supplementary Material for additional information on study design, phenotyping, genotyping, and imputation.

**Table 1.** Effective sample size of number of participants with each symptom and symptom prevalences of genome-wide association studies

		Cohort *N* effective symptom present (Sample prevalence)		
Symptom	Abbr.	Case-enriched cohorts meta	Community cohorts meta	UKB Touchscreen
1. Depressed mood	Dep	2471 (93%)	102 071 (52%)	63 695 (56%)
2. Anhedonia	Anh	4494 (90%)	98 458 (39%)	59 274 (37%)
3a. Weight loss/decrease in appetite	AppDec	10 119 (39%)	35 837 (52%)	0
3b. Weight gain/increase in appetite	AppInc	9259 (38%)	26 681 (38%)	0
4a. Insomnia	SleDec	9418 (74%)	28 741 (79%)	0
4b. Hypersomnia	SleInc	10 031 (49%)	18 377 (50%)	0
5a. Psychomotor agitation	MotoInc	10 380 (46%)	218 (3%)	0
5b. Psychomotor slowing	MotoDec	11 130 (53%)	543 (9%)	0
6. Fatigue	Fatig	3907 (91%)	25 790 (84%)	0
7. Feelings of worthlessness/guilt	Guilt	5503 (85%)	46 694 (59%)	0
8. Diminished concentration	Conc	3793 (91%)	32 827 (76%)	0
9. Recurrent thoughts of death or suicide	Sui	10 545 (65%)	50 035 (44%)	0

Case-enriched (PGC, AGDS, GenScot) and Community (ALSPAC, EstBB, UKB-MHQ) cohorts meta-analyses and UKB Touchscreen.

### Genome-wide association symptom meta-analysis

Genome-wide association study (GWAS) analyses were conducted on each symptom separately in the cohorts (PGC, AGDS, GS:SFHS, ALSPAC, EstBB, UKB-Mental Health Questionnaire) on participants who had genetic similarity with each other and with the 1000 Genomes European reference. Participants in UKB who clustered with other reference populations were not analyzed because sample sizes did not meet the threshold for LD score estimation (*N* > 5000). We also included GWAS of separate measures of depressed mood and anhedonia symptoms from the UK Biobank (UKB-Touchscreen). See Supplementary Material for more information on the individual study GWASs. We meta-analyzed the GWAS summary statistics for each symptom into ‘Case-enriched’ (PGC, AGDS, and GS:SFHS) and ‘Community’ (ALSPAC, EstBB, and UKB-MHQ) groups. We performed the meta-analyses using Ricopili (Lam et al., 2020) and calculated SNP-based heritability using LD Score Regression (LDSC) (Bulik-Sullivan et al., 2015). We assessed significant associations in the meta-analyzed summary statistics at *p* < 5 × 10^−8^/22 (the number of meta-analyses conducted) or at *p* < 5 × 10^−8^ with prior association or biological evidence at the locus.

### Confirmatory factor analysis of genetic covariance structure

We fit confirmatory genetic factor analysis models to the meta-analyzed cohort (i.e. Case-enriched and Community) and UKB Touchscreen summary statistics for each symptom using Genomic SEM (Grotzinger et al., 2019). This method uses LDSC to estimate genetic variances of and covariances among all the summary statistics. It then uses this matrix to condition structural equation models fit in lavaan (Rosseel, 2012). We first fit a common factor model, where all symptoms load on a single factor as a baseline, using symptoms with a non-negative LDSC heritability (Model ‘Depr’). To explore how sample ascertainment influenced the genetic correlations among the symptoms, we fit a series of models that captured various aspects of the sampling, measurement, and missing data processes. We then used these results to inform the construction of models that grouped the symptoms based on previous findings and diagnostic criteria. We assessed relative model fit using Akaike Information Criterion (AIC) to pick the best model and absolute model fit with Standardized Root Mean Square Residual (SRMR) to determine how well the model was capturing the genetic correlations among symptoms. We examined residual correlations to understand what aspects of symptom structure were not being captured. Factor structures are listed in online Supplementary Table S4 and illustrated in Fig. 2 and online Supplementary Figure S1.

#### Ascertainment/measurement models

The most pertinent measurement difference among the symptoms was based on the type of recruitment, so we created a two-factor model where all symptoms from the same cohorts (Case-enriched or Community) loaded together (Model ‘Case-Comm’). The next model considered the effect of the cardinal symptoms as gating items responsible for missing data patterns in UK Biobank and posited a general MDD factor that all the symptoms loaded on alongside an uncorrelated Gating factor with loadings from just the Community and UKB Touchscreen *low mood* and *anhedonia* symptoms (Model ‘Depr-Gate’). The Gating factor would therefore isolate variation associated with differences across the full non-clinical to clinical (dysphoria) spectrum. Symptoms not loading on the gating factor (i.e. those for which data are conditional on the presence of the two gating symptoms) represent variation within the more severe region of the spectrum and are thus more directly comparable to analyses of data from cases only. We then combined the Case-Community and Gating models to create a three-factor model (Model ‘Case-Comm-Gate’).

#### Symptom models

Based off the best measurement model, we then fit models that grouped symptoms into two or three factors based on previous findings from phenotypic, twin, and Genomic SEM models; and from diagnostic criteria. The first set of models grouped symptoms into Psychological and Somatic (Model ‘Psyc-Soma’); Psychological and Neurovegetative (Model ‘Psyc-Neur’); or Affective and Neurovegetative (Model ‘Affc-Neur’) factors (Elhai et al., 2012; Krause et al., 2008, 2010; Thorp et al., 2020). We further tested a model (Model ‘Cog-Mood-Neur’) with cognitive, mood, and neurovegetative symptom factors (Kendler et al., 2013).

We also fit factor models that disaggregated symptoms that involved an increasing or decreasing change (*appetite/weight, sleep,* or *psychomotor*). One such model (Model ‘CogMood-App-Leth’) was based on previous findings that identified factors for Cognitive/Mood, Appetite, and Lethargy symptoms (van Loo, Aggen, & Kendler, 2022). Finally, we considered a three-factor model (Model ‘AffCog-Melc-Atyp’) based on diagnostic criteria of melancholic and atypical depression, with the remaining symptoms loading on an Affective/Cognitive factor.

### Genetic multivariable regression

Using the best fitting symptom model, we tested how the factors were related to correlates of depression. We selected phenotypes that are known to genetically correlate with depression, including psychiatric disorders (anxiety disorder, bipolar disorder, PTSD, schizophrenia); depression defined through clinical ascertainment (MDD) and through broader or more minimal definitions (major depression); and other health, behavioral, and social phenotypes (see Supplementary Materials for list of studies). We tested whether the other phenotypes genetic correlations with each symptom factor changed after adjusting for the other factors. We did this by first fitting single regressions of a phenotype on each symptom factor. We then compared this to a multivariable regression of the phenotype on all symptom factors simultaneously. We used Benjamini–Yekutieli FDR adjustment to correct for multiple testing (Benjamini & Yekutieli, 2001).

## Results

### Genome-wide association and meta-analyses

We conducted GWAS for each symptom separately in all cohorts and meta-analyzed within sample ascertainment groups (*Case*-*enriched* cohorts: PGC, AGDS, GS:SFHS; *Community* cohorts: ALSPAC, EstBB, UKB-MHQ). We also supplemented the symptoms data with GWAS of cardinal symptoms collected at baseline in UKB (UKB-Touchscreen). (Table 1 and online Supplementary Table S1). Two associations met the stringent multiple testing burden (*p* < 5 × 10^−8^/22 = 2.27 × 10^−9^). One (rs1421085, *p* = 1.97 × 10^−16^) was an intron in *FTO* (ENSG00000140718, alpha-ketoglutarate dependent dioxygenase, a gene involved in food intake) associated with *Weight gain* in the Community cohorts. The other (rs30266, *p* = 1.94 × 10^−9^) was associated with *Anhedonia* in the Community cohorts and was an intron variant in an uncharacterized non-coding RNA gene (LOC105379109/ENSG00000251574) and previously associated with depression (Howard et al., 2019), and loneliness (Day, Ong, & Perry, 2018) (online Supplementary Table S2).

There were three additional associations that were supported by prior association studies and met the genome-wide significance threshold (*p* < 5 × 10^−8^). Two of the associations were with *Depressed mood* in the Community cohorts: rs55780333 (*p* = 1.78 × 10^−8^), an intron in COMP (ENSG00000105664, cartilage oligomeric matrix protein) also near *CRTC1* (ENSG00000105662, CREB regulated transcription coactivator 1), a gene that regulates metabolism and results in social withdrawal behaviors when knocked out in a mouse model (Breuillaud et al., 2012); and rs28665026 (*p* = 2.13 × 10^−8^) in an intron in an uncharacterized gene (LOC107986777) and associated with schizophrenia (Trubetskoy et al., 2022). An upstream variant (rs6884321, *p* = 4.27 × 10^−8^) for an uncharacterized long intergenic non-protein coding RNA (LINC01938) was associated with Community *Anhedonia* while this region was previously associated with neuroticism and MDD (Turley et al., 2018).

LDSC-estimated heritabilities were primarily in the 0.025–0.1 range. Many of the symptoms in the Case-enriched cohorts had negative heritabilities which is potentially an indication of inadequate power due to low variation. The psychomotor symptoms from the Community cohorts did not meet the sample size inclusion criteria (N_Eff_ > 5000). Out of the 12 total symptoms (taking into account directionality), 8/12 from Case-enriched meta-analysis and 10/12 from the Community meta-analysis were taken forward. The two additional cardinal symptoms from the UKB Touchscreen sample also met inclusion criteria.

### Confirmatory factor analysis

**Figure 1.**
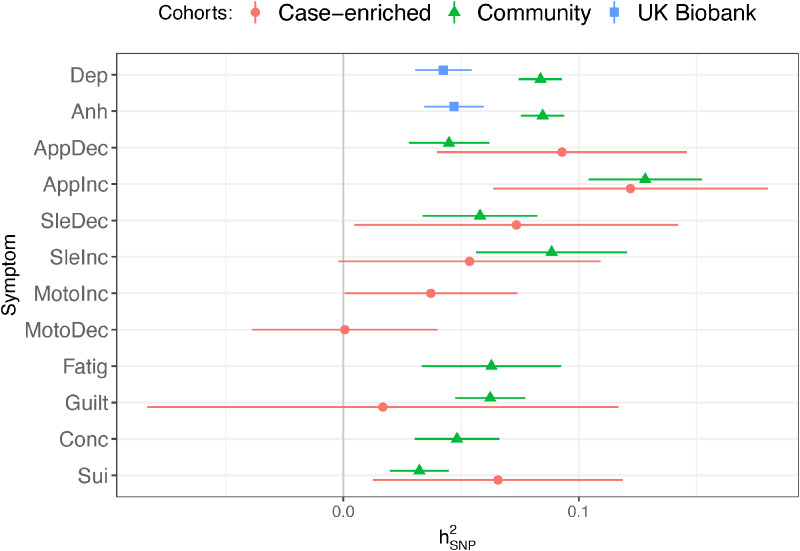
LDSC-estimated heritabilities. Heritably (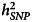) calculated on the liability scale for summary statistics that met inclusion criteria (N_Eff_ > 5000, 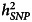 > 0). Depression symptoms abbreviations are listed in Table 1. Case-enriched = PGC + AGDS + GS:SFHS meta-analysis, Community = ALSPAC + EstBB + UKB-MHQ meta-analysis, UK Biobank = UKB-Touchscreen GWAS.

**Figure 2.**
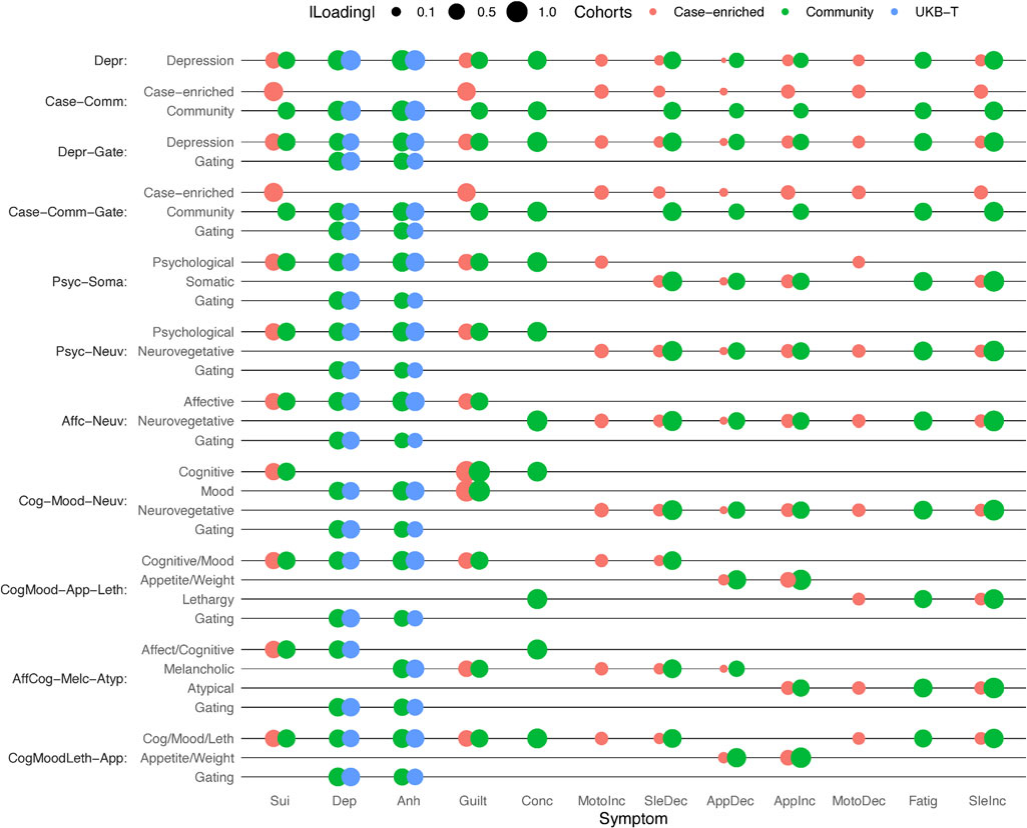
Structure and loadings of confirmatory factor models. Points representing loadings of each symptom (columns) onto each factor (rows) for confirmatory models and for the multivariate meta-analysis of well-powered GWASs to illustrate model structure, for Case-enriched (red), Community (green), and UKB Touchscreen (blue) GWASs. Size of points scaled to absolute value of factor loadings. Symptoms arranged in order so that symptoms (Affective/cognitive: Sui, Dep, :Anh, Guilt, Conc; typical somatic: MotoInc, SleDec, AppDec; and atypical somatic: AppInc, MotoDec, Fatig, SleInc) that tend to load onto the same factor are listed next to each other.

We brought forward symptoms from the Case-enriched and Community cohorts’ meta-analyses and the UKB Touchscreen assessment that had a 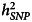 greater than 0 and sample sizes >5000 (Fig. 1, online Supplementary Table S3) for confirmatory factor analysis (Fig. 2, online Supplementary Tables S4-6, Figures S1a–n).

A common factor model (‘Depr’) of the symptoms showed poor fit (CI, 0.932, SMR = 0.169, AIC = 5355). A model (‘Case-Comm’) with separate factors for Case-enriched and Community cohort symptoms had slightly poorer fit (AIC = 5369) and yielded a genetic correlation between the two factors of *r_g_* = 0.63 ± 0.14, *p* = 1.3 × 10^−5^. An alternative model (‘Depr-Gate’) that only split off the Community and UKB-Touchscreen *Mood* and *Anhedonia* symptoms into an orthogonal factor, capturing these symptoms use as gating items in EstBB and UKB-MHQ showed substantially improved fit (AIC = 3317). A model (‘Case-Comm-Gate’) combining the sample factors with the orthogonal Gating factor showed slightly poorer fit (AIC = 3375) compared with ‘Case-Comm’ model. Therefore, we investigated the factor structure of MDD symptoms and included a gating factor accounting for symptom skip-structure in subsequent analyses.

**Figure 3.**
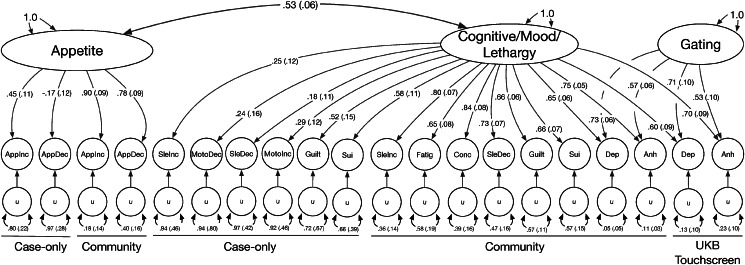
Model structural diagram. Standardized loadings (standard errors) of factors on symptoms and genetic correlations among factors for the model (*CogMoodLeth-App*) used for further analysis. Symptom abbreviations are listed in Table 1.

We tested whether models that grouped symptoms together across cohorts fit better than the factor models based on sampling methodology. The best fitting of the symptom models was the ‘CogMood-App-Leth’ model which included factors capturing Cognitive/Mood (*Depressed mood, Anhedonia, Feelings of guilt, Insomnia, Psychomotor agitation, and Suicidality*), Appetite (*Appetite/Weight increase* and *decrease*), and Lethargy (*Psychomotor slowing, Hypersomnia, Fatigue*) symptoms. Because of a high correlation (*r_g_* = 0.91) between the Cognitive/Mood and Lethargy factors, we made a model that merged these two factors (model ‘CogMoodLeth-App’; CFI = 0.968; SRMR = 0.147). The correlation between the Cognitive/Mood/Lethargy and the Appetite factors was *r_g_* = 0.53 and we brought this model forward for subsequent analysis (Fig. 3).

An inspection of the residual genetic correlations (online Supplementary Figure S3) indicated correlations between the same symptoms across the two cohorts (e.g. Case cohorts *Appetite decrease* with Community cohorts *Appetite decrease*) were not fully represented by the factor structure. We thus tested how adding residual correlations between symptoms that were well-powered enough to have been included from both cohorts (*Appetite decrease, Appetite increase, Insomnia, Hypersomnia,* and *Suicidality*) improved absolute model fit (model ‘CogMood-App-Leth [Res]’). The addition of these residual correlations lowered SRMR to 0.139 (online Supplementary Figure S1N).

### Genetic multivariable regression

We used genetic multivariable regression to test the genetic correlations of each MDD symptom factor with twelve clinically relevant phenotypes, using genome-wide summary statistics. For each external phenotype, we used Genomic SEM to fit single regressions of the phenotype onto each MDD symptom factor separately. We then fitted a multiple regression of each phenotype onto all factors to test whether a phenotype's association with each factor changed after adjusting for the other factors. We tested phenotypes against the Cognitive/Mood/Lethargy, Appetite, and Gating factors (model ‘CogMoodLeth-App’).

**Figure 4.**
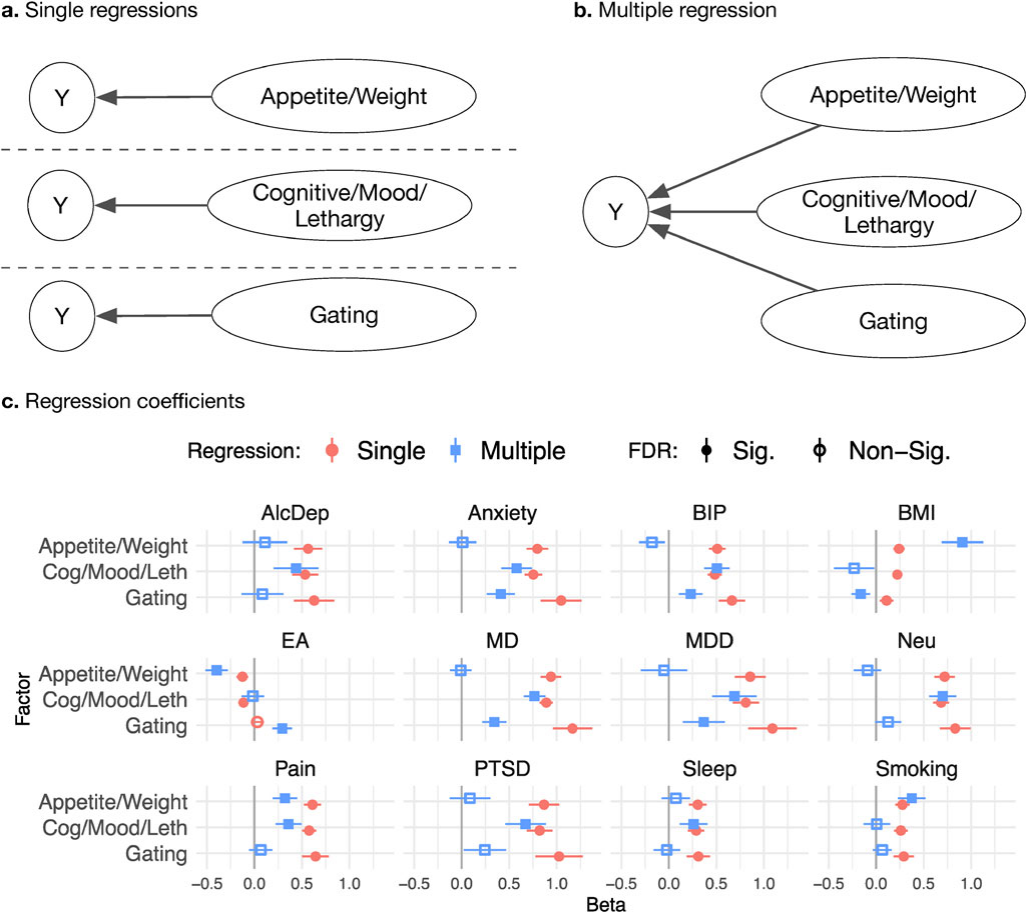
Genetic multivariable regression. (a) Model diagrams for single regressions and (b) multiple regressions of a phenotype Y on Appetite/Weight, Cognitive/Mood/Lethargy, and Gating symptom factors (symptom indicator variables omitted for clarity). (c) Single genetic regression standardized beta coefficients (green triangles) and multiple genetic regression (red circles) coefficients (point estimates plotted with 95% confidence intervals). FDR correction indicated for significant (darker shading) and non-significant (lighter shading) coefficients. Multiple regression models adjust for the other factors. AlcDep, alcohol dependence; Anxiety, anxiety disorder; BIP, bipolar disorder; BMI, body-mass index; EA, educational attainment; MD, major depression; MDD, major depressive disorder; Neu, neuroticism; Pain, chronic pain; PTSD, post-traumatic stress disorder; Sleep, long sleep duration; Smoking, cigarettes per day.

In the single regression (unadjusted) analysis, the genetic relationship of each phenotype with all the factors were significant and in the same direction, apart from educational attainment which had a negative relationship with most of the factors (at *p* < 0.0005) but a positive yet non-significant relationship with the Gating factor (Fig. 4, online Supplementary Table S7). When adjusting for the Cognitive/Mood/Lethargy and Gating factors, Appetite symptoms factor had a larger magnitude genetic correlation with BMI and educational attainment and an unchanged correlation with smoking. After adjustment, the genetic correlation of the Appetite factor with the other phenotypes was close to 0, with the exception of pain, which decreased only slightly. Adjusting for the Cognitive/Mood/Lethargy factor for the other factors did not change its genetic correlation with alcohol dependence, anxiety, bipolar disorder, major depression and MDD, neuroticism, PTSD, or long sleep duration. Genetic correlations for the Gating factor were mostly attenuated (decreasing substantially or going to 0), except that it increased for educational attainment and flipped sign for BMI.

## Discussion

We used genome-wide association data to analyze the genetic relationships among symptoms of depression based on cohort sampling and symptom content and to estimate whether the genetic factors had specific correlates with other phenotypes. We analyzed data from two sets of cohorts: Clinical cohorts that were ascertained to have depression through clinical or interview assessments or were recruited preferentially on a history of treatment for depression; and Community cohorts that were not recruited based on disease status (but for which symptom data was typically conditioned based on endorsement of cardinal gating symptoms). We conducted GWAS of major depression symptoms in each cohort then meta-analyzed within the Clinical and Community groups.

We identified loci associated with individual major depression symptoms and with a multivariate meta-analysis of a subset of well-powered symptom GWASs. Several associations from the individual symptoms meta-analysis (rs7515828, rs30266, s6884321) have been identified previously in GWAS of or unipolar depression (EFO |ID EFO_0003761) (Sollis et al., 2023) or in meta-analyses of MDD (Als et al., 2023; Howard et al., 2019; Levey et al., 2021; Wray et al., 2018). SNPs associated with *Appetite / weight increase* have primarily come up in GWAS of body mass index and related traits (Elsworth et al., 2020; Hoffmann et al., 2018; Howe et al., 2022; Yengo et al., 2018) but another SNP in the FTO gene has also been associated with atypical subtypes (Milaneschi et al., 2014).

While the low heritabilities of symptoms from the Case-enriched cohorts limited the comprehensiveness of alternative factor models that could be tested, the best fitting model did not have a separation between Case-enriched and Community cohort symptoms. The lower power in some of the symptom GWASs also do not allow for their inclusion in a multivariate meta-analysis of the ascertainment or symptom factors, as only the psychological symptoms and data from the Community cohorts were sufficiently powered for such an analysis. We also showed that model fit was substantially improved by modeling the use of cardinal symptoms (*Low mood* and *Anhedonia*) as gating items for surveys of depression symptoms. Among the models that grouped symptoms together without consideration for symptom direction, such as a split between psychological and somatic symptoms identified in previous phenotypic (Elhai et al., 2012) and genetic (Thorp et al., 2020) analyses, had worse fit than Case-enriched/Community factor models. When directional symptoms were portioned out based on diagnostic specifiers, we found that a model capturing Cognitive/Mood, Appetite, and Lethargy symptoms (van Loo et al., 2022) had the best fit among all models considered. The correlations among the factors indicated that the Cognitive/Mood and Lethargy symptoms should be grouped together, with only the Appetite symptoms making up a possibly different dimension of depression.

For the symptoms suitable for inclusion in the models that were available from both sets of cohorts (*Appetite, Sleep, Feelings of guilt,* and *Suicidality*), the Case-enriched cohorts contributed between 10 and 35% of the total effective sample size. However, the Case-enriched cohort symptoms had low loadings in both the sample-based and symptom-based models (except for the Case-enriched *Appetite/Weight* and *Suicidality* symptoms), and thus the model fit was driven primarily by capturing the structure among the Community cohort symptoms. This observation is consistent with the fact that the Clinical cohorts are more selected than the community cohorts, and that conditioning data presence on a diagnosis can induce downward bias in correlations amongst the symptoms that aggregate to form the diagnosis. Similar attenuation, albeit to a lesser degree, may be expected for items in community samples whose presence was conditioned on endorsement of cardinal symptoms. Like many recent genetic studies of depression, there is thus a need to increase the proportion of severely affected participants included in the analysis and, more specifically for understanding heterogeneity, to score symptoms in such a way as to capture more variation in severity.

A multivariable genetic regression analysis showed discriminative validity between the symptom factors, with the Appetite factor still being genetically correlated with BMI and smoking after adjusting for the other factors, and a similar pattern being observed for the Cognitive/Mood/Lethargy factor with other psychiatric phenotypes. The increase in magnitude of the genetic correlation of BMI and educational attainment with Appetite symptoms combined with the sign flip for the Gating symptom could be a part of study participation bias. However, a positive genetic correlation between increase in appetite/weight with BMI has previously been shown with PGC cohorts (Milaneschi et al., 2017) and in UKB (Badini et al., 2022), and our findings show that this result holds even when adjusting for genetic overlap with other symptoms. Yet the reliability of these findings is limited by the poor absolute fit of the models considered, which can be attributed to the proposed models all missing some aspect of the genetic structure as well as to small sample size in some of the contributing GWAS, particularly from the Case-enriched cohorts.

Our results demonstrate the challenges and insights associated with considering symptoms of depression separately. Substantial care must be taken to consider how samples are ascertained (clinical *v.* community recruitment), how symptoms are measured (the use of gating items in symptom inventories), and whether assessments of item direction (e.g. insomnia *v.* hypersomnia) are included when modeling the genetic structure of depression symptoms. However, the evaluation of direction was limited to a small subset of symptoms and did not include distinctions such as low *v.* irritable mood, or included only partial assessments, such as weight but not appetite changes being assessed in UKB. The symptoms also did not cover all diagnostic features of the atypical specifier or other sources of heterogeneity such as onset, life event exposure, or treatment outcomes (Harald & Gordon, 2012) which may have a differential biological and genetic basis (Beijers, Wardenaar, van Loo, & Schoevers, 2019; Milaneschi et al., 2020; Nguyen et al., 2022). Even the best fitting model that we tested had poor absolute fit, and thus the search for alternative models, girded by more complete data, will continue. The strongest genetic associations were between symptoms of weight/appetite change and genes linked to satiety and metabolism. This highlights the need to phenotype somatic symptoms (weight or sleep changes and fatigue) outside of the context of mental health assessments, so that their specific role in depression can be better isolated, and mirrors the larger need consider how symptoms are expressed inside and outside of a depressive episode (Kendler & Aggen, 2023). Likewise, the use of gating symptoms makes it difficult to fully capture the range of genetic risk between everyday dysphoria and differences among affected individuals. While the results support the idea that depression is heterogeneous, the genetic liability for symptom profiles and comorbidities can be captured in relatively few dimensions.

## Supporting information

Adams et al. supplementary material Adams et al. supplementary material

## Data Availability

Primary code is available from the PGC GitHub Repository (https://github.com/psychiatric-genomics-consortium/mdd-symptom-gwas/) and meta-analyzed summary statistics are available for download from the PGC website (https://www.med.unc.edu/pgc/download-results/). Individual-level PGC data is available by application to the PGC Data Access Committee (https://www.med.unc.edu/pgc/shared-methods/). Data from Estonian Biobank (https://genomics.ut.ee/en/content/estonian-biobank), UK Biobank (https://www.ukbiobank.ac.uk), and ALSPAC (http://www.bristol.ac.uk/alspac/) are available to bona fide researchers upon application. Data from AGDS is available for collaboration by contacting NGM (Nick.Martin@qimrberghofer.edu.au).

## Supplementary material

The supplementary material for this article can be found at https://doi.org/10.1017/S0033291724001880.

## Declarations

CL sits on the SAB for Myriad Neuroscience, has received consultancy fees from UCB, and speaker fees from SYNLAB. HJG has received travel grants and speaker's honoraria from Fresenius Medical Care, Neuraxpharm, Servier and Janssen Cilag as well as research funding from Fresenius Medical Care.

The authors assert that all procedures contributing to this work comply with the ethical standards of the relevant national and institutional committees on human experimentation and with the Helsinki Declaration of 1975, as revised in 2008.
