# Supplementary material for: Genome-wide meta-analysis of ascertainment and symptom structures of major depression in case-enriched and community cohorts
Source: Psychol Med. 2024 Sep 26;54(12):3459–68. doi: 10.1017/S0033291724001880 (PMC11496230; doi:10.1017/S0033291724001880)
Supplement: Adams et al. supplementary material [file S0033291724001880sup001.docx]

# **Supplementary text: Genetic structure of major depression symptoms in clinical and population cohorts**

Table of Contents

[Supplementary text: Genetic structure of major depression symptoms in clinical and population cohorts 1](#_Toc144371479)

[Cohort information 1](#_Toc144371480)

[Genotyping, QC, and GWAS 3](#_Toc144371481)

[Ethics statements 4](#_Toc144371482)

[Confirmatory factor analysis model schematics 5](#_Toc144371483)

[Figure S1. Schematic drawings of the CFA models 5](#_Toc144371484)

[Symptom genetic correlations 8](#_Toc144371485)

[Figure S1. Genetic correlations between symptoms 8](#_Toc144371486)

[Figure S3. Model implied and residual proportions of genetic correlations 8](#_Toc144371487)

[External phenotype summary statistics 10](#_Toc144371488)

[Major Depressive Disorder Working Group of the Psychiatric Genomics Consortium 11](#_Toc144371489)

[References 27](#_Toc144371490)

## Cohort information

**Psychiatric Genomics Consortium (PGC):** Data from the PGC drawn from 23 cohorts in the Wave 1 and Wave 2 datasets of the Major Depressive Disorder Working Group (Major Depressive Disorder Working Group of the Psychiatric GWAS Consortium, 2013; Wray et al., 2018). Symptoms were assessed by trained interviewers using structured diagnostic instruments and DSM checklists. Because information on symptom presence was not available for control participants in most cohorts, participants with a diagnosis of depression were selected for analysis (N = 12,821).

**Australian Genetics of Depression Study (AGDS)** (Byrne et al., 2020; Mitchell et al., 2022) is a study of depression and therapeutic response recruited using nationwide prescribing history and through publicity targeting adults who are or had ever been treated for clinical depression (N = 20,689). Symptoms experienced during the participant’s worst period of depression were assessed using the Composite International Diagnostic Interview (CIDI) Short Form (Hickie et al., 2001) and administered through an online questionnaire. Because the study was enriched for participants with a history of being diagnosed with or treated for depression, AGDS was grouped as a Clinical cohort.

**Avon Longitudinal Study of Parents and Children (ALSPAC):** Pregnant women resident in Avon, UK with expected dates of delivery between 1st April 1991 and 31st December 1992 were invited to take part in the study (Boyd et al., 2013; Fraser et al., 2013). 20,248 pregnancies have been identified as being eligible and the initial number of pregnancies enrolled was 14,541. Of the initial pregnancies, there was a total of 14,676 foetuses, resulting in 14,062 live births and 13,988 children who were alive at 1 year of age. When the oldest children were approximately 7 years of age, an attempt was made to bolster the initial sample with eligible cases who had failed to join the study originally. As a result, when considering variables collected from the age of seven onwards (and potentially abstracted from obstetric notes) there are data available for more than the 14,541 pregnancies mentioned above: The number of new pregnancies not in the initial sample (known as Phase I enrolment) that are currently represented in the released data and reflecting enrolment status at the age of 24 is 906, resulting in an additional 913 children being enrolled (456, 262 and 195 recruited during Phases II, III and IV respectively). The phases of enrolment are described in more detail in the cohort profile paper and its update. The total sample size for analyses using any data collected after the age of seven is therefore 15,447 pregnancies, resulting in 15,658 foetuses. Of these 14,901 children were alive at 1 year of age.

Of the original 14,541 initial pregnancies, 338 were from a woman who had already enrolled with a previous pregnancy, meaning 14,203 unique mothers were initially enrolled in the study. As a result of the additional phases of recruitment, a further 630 women who did not enrol originally have provided data since their child was 7 years of age. This provides a total of 14,833 unique women (G0 mothers) enrolled in ALSPAC as of September 2021.

Participants were from the original children sample (N = 13,988) with symptoms present during the last two weeks assessed using the Clinical Interview Schedule Revised (CIS-R) (Lewis et al., 1992) collected during a clinical visit at age 18. Participants were considered to have had a symptom if they reported it at the occasion.

Please note that the study website contains details of all the data that is available through a fully searchable data dictionary and variable search tool" and reference the following webpage: <http://www.bristol.ac.uk/alspac/researchers/our-data/>.

**Generation Scotland: Scottish Family Health Study (GS:SFHS)** is a study of 7,000 families recruited from the general population of Scotland (Smith et al., 2012). Participants who screened reported seeking help for emotional or psychiatric problems were administered an in-person structured interview (Fernandez-Pujals et al., 2015; Smith et al., 2012); and a subset participated in an online follow-up that included a CIDI (Composite International Diagnostic Interview) questionnaire. Symptom data was analysed on participants who met DSM criteria for depression at either time point (N = 3,493).

**Estonian Biobank (EstBB)** is a population health cohort recruited from medical practitioners in Estonia (Leitsalu et al., 2015). Participants responded to a CIDI questionnaire of depression symptoms during the Mental Health online Survey (MHoS) recontact. Participants were first screened of the presence of low mood or anhedonia and then asked about symptoms during the worst period of depression (N = 84,079).

**UK Biobank (UKB)** is a population health cohort recruited from general practitioners in the United Kingdom (Sudlow et al., 2015). Lifetime depression symptoms were assessed during online recontact and taken from the CIDI portion of the Mental Health Questionnaire (Davis et al., 2020) (UKB-MHQ, N=157,366) and from assessments of low mood and anhedonia ("ever had [symptom] for a full week") from the touchscreen questionnaire (data fields 4598 and 4631) collected at baseline and during repeat and imaging assessments (UKB Touchscreen, N=222,061). For the CIDI, low mood and anhedonia were used as gating symptoms, where participants had to endorse at least one to be asked about the other symptoms. The symptom was present if the question was endorsed as "Yes", absent if answered as "No", and missing otherwise. For the touchscreen items, the symptom was considered present if it was endorsed ("Yes") at any assessment and absent if it was responded to with "No" at all available time points.

## Genotyping, QC, and GWAS

**Psychiatric Genomics Consortium (PGC).** The analysis used data from 24 cohorts from the PGC MDD datasets that had symptom data on cases. Data was drawn from the following cohorts:

- BiDirect (bidi1)
- BOMA (boma)
- CoFams (cof3)
- PsyCoLaus (col3)
- GenRED (gens, grnd)
- GenPod/Newmeds (gep3)
- GSK (gsk2)
- Jannsen (janpy)
- MPIP/MARS (mmi2, mmo4)
- NESDA/NTR (nes1)
- QIMR (qi3c, qi6c, qio2)
- RADIANT (rad3, rage, rai2, rau2, rde4)
- Rotterdam (rot4)
- SHIP (shp0)
- STAR*D (stm2)
- TwinGene (twg2)

The genotypes were processed through Ricopili (Lam et al., 2020) with the following QC: SNP missingness < 0.05; sample missingness < 0.02; autosomal heterozygosity deviation (|Fhet|<0.2); and SNP Hardy-Weinberg equilibrium (P>10^−6^ in controls, P>10^−10^ in cases). QC'd genotypes were then imputed to the 1000 Genomes Reference Panel (The 1000 Genomes Project Consortium, 2015). Information on cohort genotyping and additional processing steps is available in (Wray et al., 2018).

**Australian Genetics of Depression Study (AGDS).** Genotyping was conducted using the Illumina Infinium Global Screening Array platform and QC'd for unknown or ambiguous map position and strand alignment, missingness >5%, HWE < 1 ×10^-6^, MAF<1%. Genotypes were imputed to HRCr1.1. Individuals were excluded with missing rate > 3%, inconsistent sex, or if deemed ancestry outliers from the European population (6 standard deviations from the first two genetic principal components from 1000 Genomes). Imputed genotype dosages were used for the analyses. GWAS was carried out in SAIGE (Zhou et al., 2018) using a generalized linear mixed model with genotyping batch and 10 PCs as covariates. Variants with MAF<1% and imputation accuracy score <0.7 were excluded.

**Avon Longitudinal Study of Parents and Children (ALSPAC).** ALSPAC children were genotyped using the Illumina HumanHap550 quad chip genotyping platforms. Individuals were excluded on the basis of gender mismatches; minimal or excessive heterozygosity; disproportionate levels of individual missingness (>3%) and insufficient sample replication (IBD < 0.8). Population stratification was assessed by multidimensional scaling analysis, removing samples that clustered outside the CEU HapMap2 population. SNPs with a minor allele frequency of < 1%, a call rate of < 95% or evidence for violations of Hardy-Weinberg equilibrium (P < 5E-7) were removed. Cryptic relatedness was measured as proportion of identity by descent (IBD > 0.1). Related subjects that passed all other quality control thresholds were retained during subsequent phasing and imputation. 9,115 subjects and 500,527 SNPs passed these quality control filters. Imputation of the target data was performed using Impute V2.2.2 against 1000 genomes reference panel (Phase 1, Version 3) (all polymorphic SNPs excluding singletons), using all 2186 reference haplotypes (including non-Europeans). This resulted in 28,699,419 SNPs, with 8,282,911 SNPs with a MAF >0.01 and info score of >0.8. Analysis were conducted using SNPTEST v2.5.2, adjusting for sex and the first 10 principal components of ancestry.

**Generation Scotland (GS:SFHS).** GWAS data was obtained using the Illumina OmniExpress array, and imputed using the Haplotype Research Consortium (HRC) dataset. Further details of methods here https://pubmed.ncbi.nlm.nih.gov/28270201/. GWAS was conducted in regenie with 4 PCs removing SNPs with MAC < 100, genotype missingness > 10%, INFO < 0.1, and HWE p > 1e-15.

**Estonian Biobank (EstBB).** The samples from the Estonian Biobank have been genotyped at the Genotyping Core Facility of the Institute of Genomics, University of Tartu using the Global Screening Array (GSAv1.0, GSAv2.0, and GSAv2.0_EST) from Illumina. Altogether 155,772 samples have been genotyped and PLINK format files exported using GenomeStudio v2.0.4. Individuals were excluded from the analysis if their call-rate was <95% or if the sex defined based on heterozygosity of the X chromosome did not match the sex in the phenotype data. Variants were excluded if the call-rate was < 95% and HWE p-value <1e-4 (autosomal variants only). Variant positions were updated to genome build 37 and all alleles were switched to the TOP strand using tools and reference files provided at https://www.well.ox.ac.uk/~wrayner/strand/. After QC the dataset contained 154,201 samples for imputation. Before imputation variants with MAF<1% and indels were removed. Prephasing was done using the Eagle v2.3 software. The number of conditioning haplotypes Eagle2 uses when phasing each sample was set to: --Kpbwt=20000. Imputation was done using Beagle v.28Sep18.793 with effective population size ne=20,000. An Estonian population specific imputation reference of 2,297 WGS samples was used. The analysis was performed using the SAIGE software, including related individuals and adjusting for the first 10 principal components (PCs) of the genotype matrix, as well as for birth year, birth year squared and sex.

**UK Biobank (UKB).** Imputed genotypes were analysed from the version 3 release (Bycroft et al., 2018). Imputed genotypes were QC'd to INFO >= 0.1, MAC >= 100, HWE P > 1e-10, max alleles = 2, and duplicate markers removed. Association analysis was performed as a logistic regression in Plink2 (Chang et al., 2015) with genotyping array and 20 PCs as covariates.

**GWAS Meta-analysis and LD Score.** For input into LDSC we set the sample size equal to the sum of effective sample sizes of each cohort in the meta-analysis and then specified sample prevalences of 50% (Grotzinger, Fuente, et al., 2022). Symptoms' population prevalences were estimated for the Clinical cohorts by multiplying the observed sample prevalence by the prevalence of MDD (15%) and for the Community cohorts by multiplying by the proportion of participants in the UKB MHQ sample who were positive on either one the gating symptoms.

## Ethics statements

Ethical approval was obtained from the ALSPAC Ethics and Law Committee and the local research ethics committees (project number B3118). Consent for biological samples has been collected in accordance with the Human Tissue Act (2004).

All participants in AGDS provided informed consent that they had read and understood the study information sheets and to confirm that they would be willing to provide a saliva sample for genotyping and downstream generic analyses. All study protocols were approved by the QIMR Berghofer Medical Research Institute Human Research Ethics Committee - approval numbers P2118, P1309 and P2304.

The activities of the EstBB are regulated by the Human Genes Research Act, which was adopted in 2000 specifically for the operations of the EstBB. Individual-level data analysis in the EstBB was carried out under ethical approvals [1.1-12/2860 & 1.1-12/624] from the Estonian Committee on Bioethics and Human Research (Estonian Ministry of Social Affairs), using data according to the release application [3-10/GI-28207] from the Estonian Biobank.

Ethical approval for the GS:SFHS data collection was obtained from the Tayside Committee on Medical Research Ethics A (ref 05/S1401/89). Generation Scotland is currently approved as a Research Tissue Bank by the East of Scotland Research Ethics Service (ref 20/ES/0021).

UK Biobank received ethical approval from the Research Ethics Committee (reference 11/NW/0382).

## Confirmatory factor analysis model schematics

### Figure S1. Schematic drawings of the CFA models

Schematics to illustrate factor structures of the models that were tested. Symptoms are grouped by those from Case-enriched/Clinical cohorts, those from Community cohorts, and those from the UKB Touchscreen. Residual variances omitted for clarity. See also Supplementary Tables S4 for factor structures.

Symptom abbreviations:

- Dep: Depressed mood
- Anh: Anhedonia
- AppDec: Weight loss / decrease in appetite
- AppInc: Weight gain / increase in appetite
- SleDec: Insomnia
- SleInc: Hypersomnia
- MotoInc: Psychomotor agitation
- MotoDec: Psychomotor slowing
- Fatig: Fatigue
- Guilt: Feelings of worthlessness / guilt
- Conc: Diminished concentration
- Sui: Recurrent thoughts of death or suicide

### Figure S1. Diagrams of CFA models with parameter estimates.

See also Supplementary Tables S6 for parameter estimates.

Figure S1a: Model "Depr": Common factor

Figure S1b: Model "Case-Comm": Case-enriched (Clinical) and community factors

Figure S1c: Model "Depr-Gate": Gating measurement factor

Figure S1d: Model "Case-Comm-Gate": Clinical-Community-Gating factors

Figure S1e: Model "Psyc-Soma": Psychological-Somatic

Figure S1f: Model "Psyc-Neuv": Psychological- Neurovegetative

Figure S1g: Model "Affc-Neuv": Affective-Neurovegetative

Figure S1h: Model "Cog-Mood-Neuv": Cognitive-Mood-Neurovegetative

Figure S1i: Model "CogMood-App-Leth": Cognitive/Mood-Appetite-Lethargy

Figure S1j: Model "AffCog-Melc-Atyp": Affective/Cognitive-Melancholic-Atypical

Figure S1m: Model "CogMoodLeth-App Cog/Mood/Lethargy-Appetite-

Figure S1n: Model "CogMoodLeth-App [Res]": Cog/Mood/Lethargy-Appetite-Lethargy with residual correlations

## Symptom genetic correlations

### Figure S2. Genetic correlations between symptoms


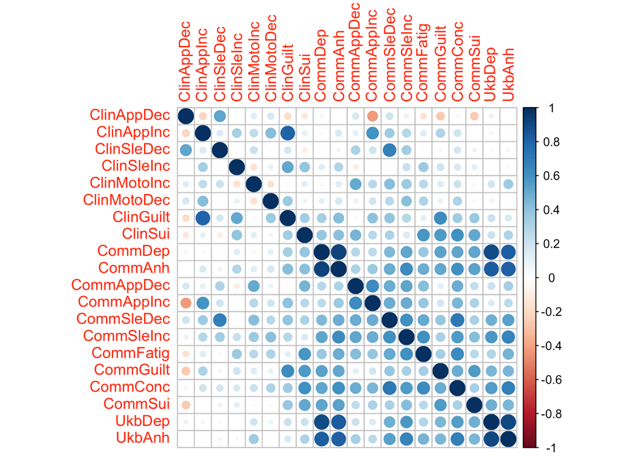


###

### Figure S3. Model implied and residual proportions of genetic correlations

Variance and covariances scaled by total genetic variance of each symptom.

Figure S3a. Model implied proportions of genetic correlations for Case-Community-Gating factors (Model "Case-Comm-Gate")


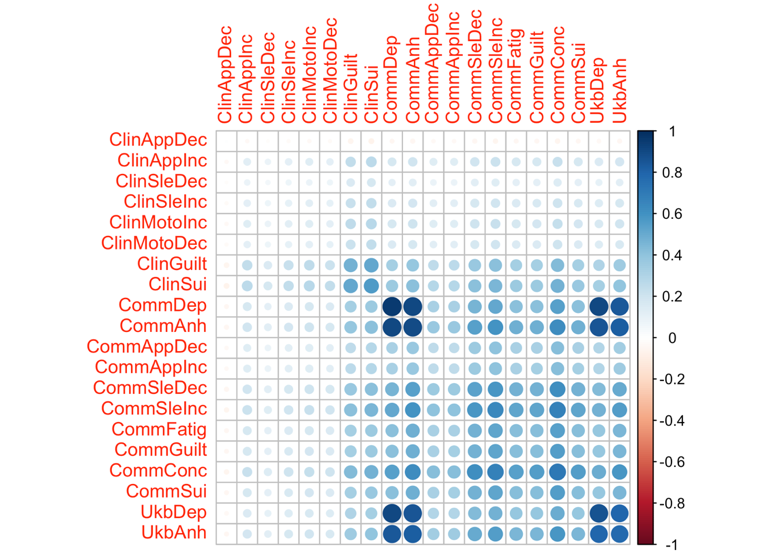


Figure S3b. Model residual proportions of genetic correlations for Case-Community-Gating factors (Model Case-Comm-Gate)


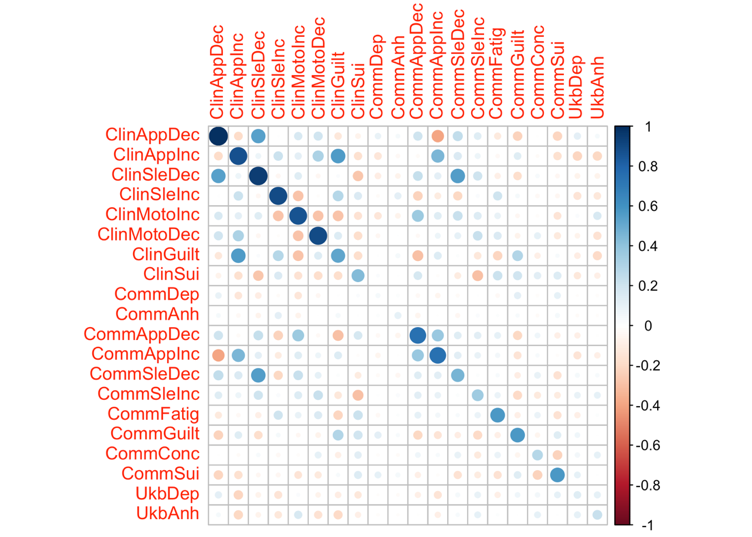


Figure S3c. Model implied proportions of genetic correlations for Cognitive/Mood-Appetite-Lethargy factors (Model "CogMood-App-Leth")


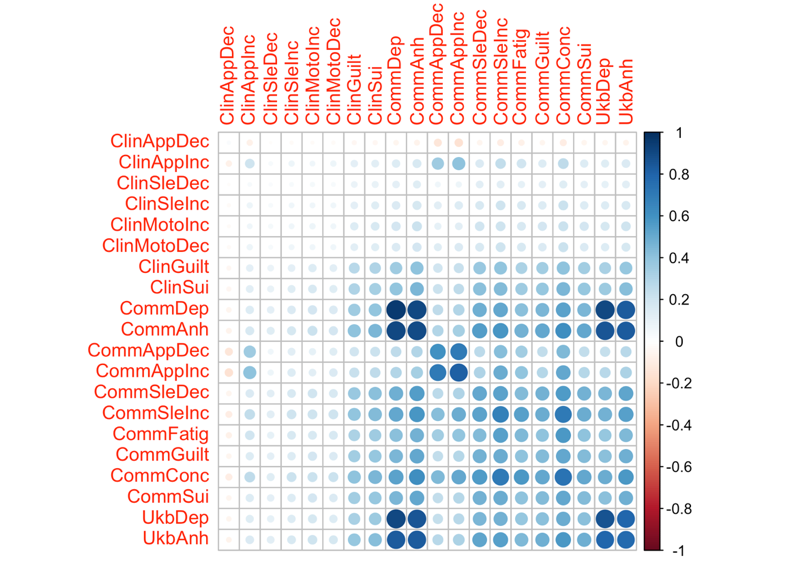


Figure S3d. Model residual proportions of genetic correlations for Cognitive/Mood-Appetite-Lethargy factors (Model "CogMood-App-Leth")


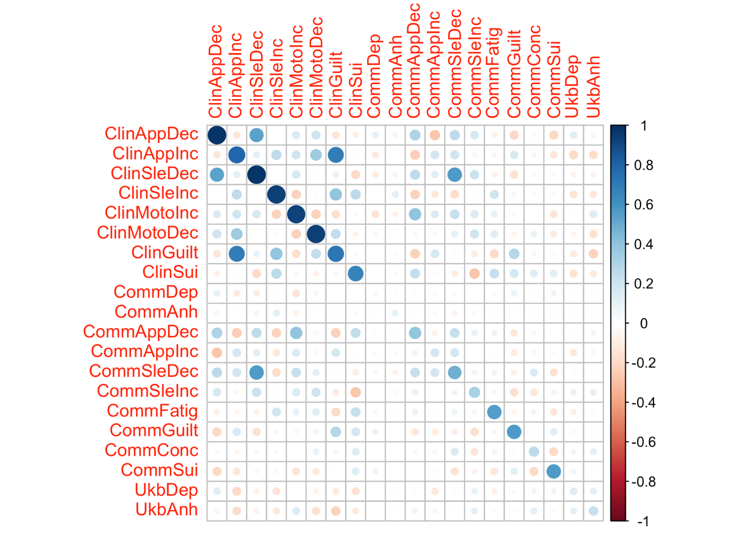


## External phenotype summary statistics

For the genetic multiple regression analysis, we used the following summary statistics:

- Alcohol dependence (Walters et al., 2018)
- Anxiety (Grotzinger, Mallard, et al., 2022)
- Bipolar disorder (Mullins et al., 2021)
- Body mass index (Pulit et al., 2019)
- Educational attainment (Okbay et al., 2022)
- Major depression (Als et al., 2023)
- Major depressive disorder (Wray et al., 2018)
- Neuroticism (Nagel et al., 2018)
- Pain (multisite chronic pain) (Johnston et al., 2019)
- Post-traumatic stress disorder (Nievergelt et al., 2019)
- Sleep (long sleep duration) (Dashti et al., 2018)
- Smoking (cigarettes per day) (Liu et al., 2019)

## Major Depressive Disorder Working Group of the Psychiatric Genomics Consortium

Mark J Adams 1

Fabian Streit 2

Swapnil Awasthi 3

Brett N Adey 4

Karmel W Choi 5, 6

V Kartik Chundru 7

Jonathan RI Coleman 4, 8

Jerome C Foo 2

Olga Giannakopoulou 9

Alisha S M Hall 2, 10

Jens Hjerling-Leffler 11

David M Howard 4

Christopher Hübel 4, 12, 13

Alex S F Kwong 1, 14

Bochao Danae Lin 15

Xiangrui Meng 9

Guiyan Ni 16

Oliver Pain 17

Gita A Pathak 18, 19

Eva C Schulte 20, 21, 22, 23

Jackson G Thorp 24

Alicia Walker 16

Shuyang Yao 25

Jian Zeng 16

Johan Zvrskovec 4, 8

Dag Aarsland 26

Ky'Era V Actkins 27

Mazda Adli 3, 28

Esben Agerbo 12, 29, 30

Mareike Aichholzer 31

Tracy M Air 32

Allison Aiello 33

Thomas D Als 30, 34, 35

Evelyn Andersson 36

Till F M Andlauer 37, 38

Volker Arolt 39

Helga Ask 40, 41

Sunita Badola 42

Clive Ballard 43

Karina Banasik 44

Nicholas J Bass 9

Aartjan T F Beekman 45

Sintia Belangero 46

Elisabeth B Binder 38, 47

Ottar Bjerkeset 48, 49

Gyda Bjornsdottir 50

Julia Boberg 36

Sigrid Børte 51, 52, 53

Emma Bränn 54

Alice Braun 55

Thorsten Brodersen 56

Søren Brunak 44

Mie T Bruun 57

Pichit Buspavanich 58, 59

Jonas Bybjerg-Grauholm 60, 61

Enda M Byrne 62

Archie Campbell 63, 64

Megan L. Campbell 65

Enrique Castelao 66

Jorge Cervilla 67, 68

Boris Chaumette 69

Chia-Yen Chen 70

Zhengming Chen 71, 72

Sven Cichon 73, 74, 75, 76

Lucía Colodro-Conde 24

Anne Corbett 43

Elizabeth C Corfield 40, 77

Baptiste Couvy-Duchesne 78

Nick Craddock 79, 80

Udo Dannlowski 39

Gail Davies 81

EJC de Geus 82

Ian J Deary 81

Franziska Degenhardt 76, 83

Abbas Dehghan 84, 85

J Raymond DePaulo 86

Michael Deuschle 87

Maria Didriksen 88

Khoa Manh Dinh 89

Nese Direk 90

Srdjan Djurovic 91, 92

Anna R Docherty 93, 94, 95

Katharina Domschke 96

Joseph Dowsett 88

Ole Kristian Drange 49, 97, 98, 99

Erin C Dunn 6, 100

Gudmundur Einarsson 50

Thalia C Eley 4

Samar S M Elsheikh 101

Jan Engelmann 102

Michael E Benros 60, 103, 104

Christian Erikstrup 89

Valentina Escott-Price 80

Chiara Fabbri 4, 105

Yu Fang 106

Sarah Finer 107

Josef Frank 2

Robert C Free 108

He Gao 109

Michael Gill 110

Maria Gilles 87

Fernando S Goes 86

Scott Douglas Gordon 24

Jakob Grove 30, 34, 35, 111

Daniel F Gudbjartsson 50, 112

Blanca Gutierrez 67, 68

Tim Hahn 39

Lynsey S Hall 80

Thomas F Hansen 44, 60, 113

Magnus Haraldsson 114

Catherina A Hartman 115

Alexandra Havdahl 40

Caroline Hayward 116

Stefanie Heilmann-Heimbach 76

Stefan Herms 74, 76

Ian B Hickie 117

Henrik Hjalgrim 118

Per Hoffmann 74, 76

Georg Homuth 119

Carsten Horn 120

Jouke-Jan Hottenga 82

David M Hougaard 60, 61

Iiris Hovatta 121

Qin Qin Huang 7

Floris Huider 82

Karen A Hunt 122

Marcus Ising 123

Erkki Isometsä 124

Rick Jansen 45

Yunxuan Jiang 125

Ian Jones 80

Lisa A Jones 126

Lina Jonsson 127

Robert Karlsson 25

Siegfried Kasper 128

Kenneth S Kendler 129

Ronald C Kessler 130

Stefan Kloiber 101, 123, 131, 132

James A Knowles 133

Nastassja Koen 65

Julia Kraft 55

Henry R Kranzler 134, 135

Kristi Krebs 136

Theodora Kunovac Kallak 137

Zoltán Kutalik 138, 139, 140

Elisa Lahtela 141

Margit Hørup Larsen 88

Eric J Lenze 142

Daniel F Levey 143, 144

Melissa Lewins 1

Glyn Lewis 9

Liming Li 145, 146

Kuang Lin 71

Penelope A Lind 24

Donald J MacIntyre 1, 147, 148

Dean F MacKinnon 86

Wolfgang Maier 149

Victoria S Marshe 101, 150

Hamdi Mbarek 82

Peter McGuffin 4

Sarah E Medland 24

Susanne Meinert 39, 151

Susan Mikkelsen 89

Christina Mikkelsen 88, 152

Yuri Milaneschi 45

Iona Y Millwood 71, 72

Brittany L Mitchell 24

Esther Molina 67, 153

Francis M Mondimore 86

Preben Bo Mortensen 12, 29, 30

Benoit H Mulsant 101, 131

Joonas Naamanka 121

Jake M Najman 154

Matthias Nauck 155, 156

Igor Nenadi? 157

Kasper R Nielsen 158

Ilja M Nolte 159

Merete Nordentoft 60, 103, 104

Markus M Nöthen 76

Mette Nyegaard 30, 160, 161, 162

Michael C O'Donovan 80

Asmundur Oddsson 50

Catherine M Olsen 163, 164

Hogni Oskarsson 165

Sisse Rye Ostrowski 88, 166

Vanessa K Ota 46

Michael J Owen 80

Richard Packer 167

Teemu Palviainen 141

Pedro M Pan 168

Carlos N Pato 169

Michele T Pato 169

Nancy L Pedersen 25

Ole Birger Pedersen 170

Roseann E Peterson 129, 171

Wouter J Peyrot 45

James B Potash 86

Martin Preisig 66

Jorge A Quiroz 172

Charles F Reynolds III 173

John P Rice 142

Giovanni A Salum 174

Robert A Schoevers 175, 176

Andrew Schork 30, 177, 178

Thomas G Schulze 2, 21, 86, 179, 180

Tabea S Send 87

Jianxin Shi 181

Engilbert Sigurdsson 114

Kritika Singh 27

Grant C B Sinnamon 182

Lea Sirignano 2

Olav B Smeland 183, 184

Daniel J Smith 185

Erik Sørensen 88

Sundararajan Srinivasan 186

Hreinn Stefansson 50

Kari Stefansson 50, 187

Dan J. Stein 188

Frederike Stein 189

André Tadic 102, 190

Henning Teismann 191

Alexander Teumer 192

Anita Thapar 80, 193

Pippa A Thomson 64

Lise Wegner Thørner 88

Apostolia Topaloudi 194

Ioanna Tzoulaki 84, 85, 195

Monica Uddin 196

André G Uitterlinden 197

Henrik Ullum 88, 198, 199

Daniel Umbricht 200

Robert J Ursano 201

Sandra Van der Auwera 202

David A van Heel 122

Albert M van Hemert 203

Abirami Veluchamy 186

Alexander Viktorin 25

Henry Völzke 192

Agaz Wani 196

G Bragi Walters 50

Robin G Walters 71, 72

Sylvia Wassertheil-Smoller 204

Myrna M Weissman 205, 206

Jürgen Wellmann 191

David C Whiteman 163

Derek Wildman 196

Gonneke Willemsen 82

Alexander T Williams 167

Bendik S Winsvold 51, 52, 207

Stephanie H Witt 2

Ying Xiong 25

Lea Zillich 2

John-Anker Zwart 51, 52, 53

23andMe Research Team 125

Estonian Biobank Research Team 136

HUNT All-In Psychiatry 208

China Kadoorie Biobank Collaborative Group 209

Genes & Health Research Team 210

Ole A Andreassen 183, 184, 211

Bernhard T Baune 212, 213, 214

Klaus Berger 191

Dorret I Boomsma 82

Anders D Børglum 30, 34, 35

Gerome Breen 4, 8

Na Cai 215, 216, 217

Hilary Coon 94

William E Copeland 218

Byron Creese 43

Lea K Davis 27

Eske M Derks 24

Enrico Domenici 219

Paul Elliott 84, 85, 195, 220

Andreas J Forstner 73, 76

Micha Gawlik 221

Joel Gelernter 19, 143, 222

Hans J Grabe 202

Steven P Hamilton 223

Kristian Hveem 224, 225, 226

Catherine John 167, 227

Jaakko Kaprio 141

Tilo Kircher 157

Marie-Odile Krebs 228

Karoline Kuchenbaecker 9, 71

Mikael Landén 25, 127

Kelli Lehto 136

Douglas F Levinson 229

Qingqin S Li 230

Klaus Lieb 102

Yi Lu 25

Susanne Lucae 123

Jurjen J Luykx 15, 231

Patrik K Magnusson 25

Nicholas G Martin 24

Hilary C Martin 7

Andrew McQuillin 9

Christel M Middeldorp 62, 232

Lili Milani 136

Ole Mors 30, 233

Daniel J Müller 101, 131, 132, 234

Bertram Müller-Myhsok 38, 235, 236

Albertine J Oldehinkel 115

Sara A Paciga 237

Colin NA Palmer 186

Peristera Paschou 194

Brenda WJH Penninx 45

Roy H Perlis 5, 6, 238

Giorgio Pistis 66

Renato Polimanti 18, 19

David J Porteous 64

Danielle Posthuma 239, 240

Ted Reichborn-Kjennerud 40

Andreas Reif 31

Frances Rice 80, 241

Roland Ricken 3

Marcella Rietschel 2

Margarita Rivera 67, 242

Christian Rück 243

Catherine Schaefer 244

Srijan Sen 106, 245

Alessandro Serretti 105

Alkistis Skalkidou 137

Jordan W Smoller 5, 246, 247

Frederike Stein 189

Murray B Stein 248, 249, 250

Patrick F Sullivan 25, 251

Martin Tesli 40

Thorgeir E Thorgeirsson 50

Henning Tiemeier 252, 253

Nicholas J Timpson 14

Rudolf Uher 254

Jens R Wendland 42

Thomas Werge 60, 177, 199, 255, 256

Naomi R Wray 16, 257

Stephan Ripke 3, 246

Cathryn M Lewis 4, 258

Andrew M McIntosh 1, 25

1, Division of Psychiatry, University of Edinburgh, Edinburgh, UK

2, Department of Genetic Epidemiology in Psychiatry, Central Institute of Mental Health, Medical Faculty Mannheim, Heidelberg University, Mannheim, BW, DE

3, Department of Psychiatry and Psychotherapy, Charité – Universitätsmedizin Berlin, Berlin, BE, DE

4, Social, Genetic and Developmental Psychiatry Centre, King's College London, London, UK

5, Department of Psychiatry, Massachusetts General Hospital, Boston, MA, US

6, Department of Psychiatry, Harvard Medical School, Boston, MA, US

7, Human Genetics, Wellcome Sanger Institute, Hinxton, UK

8, NIHR Maudsley Biomedical Research Centre, King's College London, London, UK

9, Division of Psychiatry, University College London, London, UK

10, Department of Clinical Medicine, Aarhus University, Aarhus, DK

11, Department of Medical Biochemistry and Biophysics, Karolinska Institutet, Stockholm, SE

12, National Centre for Register-based Research, Aarhus University, Aarhus, DK

13, Department of Pediatric Neurology, Charité – Universitätsmedizin Berlin, Berlin, BE, DE

14, MRC Integrative Epidemiology Unit, University of Bristol, Bristol, UK

15, Department of Psychiatry and Neuropsychology, School for Mental Health and Neuroscience, Maastricht University Medical Centre, Maastricht, NL

16, Institute for Molecular Bioscience, University of Queensland, Brisbane, QLD, AU

17, Maurice Wohl Clinical Neuroscience Institute, Department of Basic and Clinical Neuroscience, King's College London, London, UK

18, Veterans Affairs Connecticut Healthcare System, West Haven, CT, US

19, Department of Psychiatry, Yale University School of Medicine, New Haven, CT, US

20, Department of Psychiatry, University of Munich, Munich, BY, DE

21, Institute of Psychiatric Phenomics and Genomics, University of Munich, Munich, BY, DE

22, Department of Psychiatry and Psychotherapy, University Hospital Bonn, Medical Faculty, University of Bonn, Bonn, DE

23, Institute of Human Genetics, University Hospital Bonn, Medical Faculty, University of Bonn, Bonn, DE

24, Mental Health and Neuroscience, QIMR Berghofer Medical Research Institute, Brisbane, QLD, AU

25, Department of Medical Epidemiology and Biostatistics, Karolinska Institutet, Stockholm, SE

26, Old Age Psychiatry, King's College London, London, UK

27, Department of Medicine, Division of Genetic Medicine, Vanderbilt University Medical Center, Nashville, TN, US

28, Department of Psychiatry and Psychotherapy, Fliedner Klinik Berlin, Berlin, BE, DE

29, Centre for Integrated Register-based Research, Aarhus University, Aarhus, DK

30, iPSYCH, The Lundbeck Foundation Initiative for Integrative Psychiatric Research, Aarhus, DK

31, Department of Psychiatry, Psychosomatic Medicine and Psychotherapy, Goethe University Frankfurt - University Hospital, Frankfurt am Main, DE

32, Discipline of Psychiatry, University of Adelaide, Adelaide, SA, AU

33, Department of Epidemiology, Columbia University Mailman School of Public Health, New York, NY, US

34, Department of Biomedicine and Centre for Integrative Sequencing, iSEQ, Aarhus University, Aarhus, DK

35, Center for Genomics and Personalized Medicine, Aarhus University, Aarhus, DK

36, Department of Clinical Neuroscience, Karolinska Institutet,, SE

37, Department of Neurology, Klinikum rechts der Isar, Technical University of Munich, Munich, BY, DE

38, Department of Translational Research in Psychiatry, Max Planck Institute of Psychiatry, Munich, BY, DE

39, Institute for Translational Psychiatry, University of Münster, Münster, NRW, DE

40, Department of Mental Disorders, Norwegian Institute of Public Health, Oslo, NO

41, PROMENTA Research Center, Department of Psychology, University of Oslo, Oslo, NO

42, Research and Development, Takeda Pharmaceutical Company Limited, Cambridge, MA, US

43, Faculty of Health and Life Sciences, University of Exeter, Exeter, UK

44, Novo Nordisk Center for Protein Research, Department of Health Sciences, University of Copenhagen, Copenhagen, DK

45, Department of Psychiatry, Amsterdam Public Health and Amsterdam Neuroscience, Amsterdam UMC, Vrije Universiteit Amsterdam, Amsterdam, NL

46, Morphology and Genetics, Universidade Federal de Sao Paulo, Sao Paulo, SP, BR

47, Department of Psychiatry and Behavioral Sciences, Emory University School of Medicine, Atlanta, GA, US

48, Faculty of Nursing and Health Sciences, NORD University, Levanger, NO

49, Department of Mental Health, Faculty of Medicine and Health Sciences, Norwegian University of Science and Technology (NTNU), Trondheim, TRD, NO

50, deCODE Genetics / Amgen, Reykjavik, IS

51, K. G. Jebsen Center for Genetic Epidemiology, Department of Public Health and Nursing, Faculty of Medicine and Health Sciences, Norwegian University of Science and Technology (NTNU), Trondheim, TRD, NO

52, Department of Research and Innovation, Division of Clinical Neuroscience, Oslo University Hospital, Oslo, NO

53, Institute of Clinical Medicine, Faculty of Medicine, University of Oslo, Oslo, NO

54, Institute of Environmental Medicine, Unit of Integrative Epidemiology, Karolinska Institutet, Stockholm, SE

55, Department of Psychiatry and Psychotherapy, Charité – Universitätsmedizin Berlin, Berlin, DE

56, Department of Clinical Immunology, Roskilde University/Næstved Hospital, Roskilde, DK

57, Department of Clinical Immunology, Odense University Hospital, Odense, DK

58, Department of Psychiatry, Psychotherapy and Psychosomatics, Brandenburg Medical School Theodor Fontane, Neuruppin, BB, DE

59, Department of Psychiatry and Psychotherapy, Gender Research in Medicine, Institute of Sexology and Sexual Medicine, Charité – Universitätsmedizin Berlin, Berlin, BE, DE

60, iPSYCH, The Lundbeck Foundation Initiative for Integrative Psychiatric Research, Copenhagen, DK

61, Center for Neonatal Screening, Department for Congenital Disorders, Statens Serum Institut, Copenhagen, DK

62, Child Health Research Centre, University of Queensland, Brisbane, QLD, AU

63, Centre for Medical Informatics, Usher Institute, University of Edinburgh, Edinburgh, UK

64, Centre for Genomic & Experimental Medicine, Institute for Genetics and Cancer, University of Edinburgh, Edinburgh, UK

65, Department of Psychiatry and Mental Health, University of Cape Town, Cape Town, SA

66, Department of Psychiatry, Lausanne University Hospital and University of Lausanne, Prilly, VD, CH

67, Instituto de Investigación Biosanitaria ibs.GRANADA, Granada, ES

68, Department of Psychiatry, Faculty of Medicine and Institute of Neurosciences, Biomedical Research Centre (CIBM), University of Granada, Granada, ES

69, Université de Paris Cité, INSERM U1266, Institute of Psychiatry and Neuroscience of Paris, GHU Paris Psychiatry and Neuroscience, Paris, FR

70, Translational Biology, Biogen, Cambridge, MA, US

71, Nuffield Department of Population Health, University of Oxford, Oxford, UK

72, MRC Population Health Research Unit, University of Oxford, Oxford, UK

73, Institute of Neuroscience and Medicine (INM-1), Research Center Juelich, Juelich, DE

74, Human Genomics Research Group, Department of Biomedicine, University of Basel, Basel, CH

75, Institute of Medical Genetics and Pathology, University Hospital Basel, University of Basel, Basel, CH

76, Institute of Human Genetics, University of Bonn, School of Medicine & University Hospital Bonn, Bonn, DE

77, Nic Waals Institute, Lovisenberg Diakonale Hospital, Oslo, NO

78, Centre for Advanced Imaging, University of Queensland, Saint Lucia, QLD, AU

79, Psychological Medicine, Cardiff University, Cardiff, WLS, UK

80, Centre for Neuropsychiatric Genetics and Genomics, Cardiff University, Cardiff, WLS, UK

81, The Lothian Birth Cohorts, University of Edinburgh, Edinburgh, UK

82, Department of Biological Psychology & Amsterdam Public Health Research Institute, Vrije Universiteit Amsterdam, Amsterdam, NL

83, Department of Child and Adolescent Psychiatry, Psychosomatics and Psychotherapy, University Hospital Essen, Unversity of Duisburg-Essen, Duisburg, DE

84, MRC Centre for Environment and Health, School of Public Health, Imperial College London, London, UK

85, Imperial College Dementia Research Institute, Imperial College London, London, UK

86, Department of Psychiatry and Behavioral Sciences, Johns Hopkins University School of Medicine, Baltimore, MD, US

87, Department of Psychiatry and Psychotherapy, Research Group Stress Related Disorders, Central Institute of Mental Health, Medical Faculty Mannheim, Heidelberg University, Mannheim, BW, DE

88, Department of Clinical Immunology, Copenhagen University Hospital, Rigshospitalet, Copenhagen, CPH, DK

89, Department of Clinical Immunology, Aarhus University Hospital, Aarhus, DK

90, Department of Psychiatry, Istanbul University, Istanbul, TR

91, Department of Medical Genetics, Oslo University Hospital, Oslo, OSL, NO

92, NORMENT, Department of Clinical Science, University of Bergen, Bergen, NO

93, Virginia Institute for Psychiatric & Behavioral Genetics, Virginia Commonwealth University, Richmond, VA, US

94, Psychiatry Department / Huntsman Mental Health Institute, University of Utah School of Medicine, Salt Lake City, UT, US

95, Center for Genomic Research, University of Utah School of Medicine, Salt Lake City, UT, US

96, Department of Psychiatry and Psychotherapy, Medical Center, University of Freiburg, Faculty of Medicine, University of Freiburg, Freiburg, DE

97, Division of Mental Health Care, St. Olavs Hospital, Trondheim University Hospital, Trondheim, TRD, NO

98, Department of Psychiatry, Sørlandet Hospital, Kristiansand, AG, NO

99, University of Oslo, NORMENT Centre, Institute of Clinical Medicine, Oslo, OSL, NO

100, Center for Genomic Medicine, Massachusetts General Hospital, Boston, MA, US

101, Centre for Addiction and Mental Health, Toronto, ON, CA

102, Department of Psychiatry and Psychotherapy, University Medical Center of the Johannes Gutenberg University Mainz, Mainz, DE

103, Mental Health Center Copenhagen, Mental Health Services Capital Region of Denmark, Copenhagen, DK

104, Faculty of Health Science, Department of Clinical Medicine, University of Copenhagen, Copenhagen, DK

105, Department of Biomedical and Neuromotor Sciences, University of Bologna, Bologna, IT

106, Michigan Neuroscience Institute, University of Michigan, Ann Arbor, MI, US

107, Wolfson Institute of Population Health, Queen Mary University of London, London, UK

108, School of Computing and Mathematical Sciences, University of Leicester, Leicester, UK

109, Department of Epidemiology and Biostatistics, Imperial College London, London, UK

110, Discipline of Psychiatry, School of Medicine, Trinity College Dublin, Dublin, IE

111, Bioinformatics Research Centre, Aarhus University, Aarhus, DK

112, School of Engineering, University of Iceland, Reykjavik, IS

113, Danish Headache Centre, Department of Neurology, Rigshospitalet, Glostrup, DK

114, Faculty of Medicine, Department of Psychiatry, University of Iceland, Reykjavik, IS

115, Department of Psychiatry, University of Groningen, University Medical Center Groningen, Groningen, NL

116, MRC Human Genetics Unit, Institute for Genetics and Cancer, University of Edinburgh, Edinburgh, UK

117, Brain and Mind Centre, University of Sydney, Sydney, NSW, AU

118, Department of Epidemiology Research, Statens Serum Institut, Copenhagen, DK

119, Interfaculty Institute for Genetics and Functional Genomics, Department of Functional Genomics, University Medicine Greifswald, Greifswald, MV, DE

120, Roche Pharmaceutical Research and Early Development, Pharmaceutical Sciences, Roche Innovation Center Basel, F. Hoffmann-La Roche Ltd, Basel, CH

121, SleepWell Research Program and Department of Psychology and Logopedics, University of Helsinki, Helsinki, FI

122, Blizard Institute, Barts and the London School of Medicine and Dentistry, Queen Mary University of London, London, UK

123, Max Planck Institute of Psychiatry, Munich, BY, DE

124, Department of Psychiatry, University of Helsinki, Helsinki, FI

125, 23andMe Research Team, 23andMe, Inc., Sunnyvale, CA, US

126, Department of Psychological Medicine, University of Worcester, Worcester, UK

127, Institution of Neuroscience and Physiology, University of Gothenburg, Gothenburg, SE

128, Department of Psychiatry and Psychotherapy, Medical University of Vienna, Vienna, AT

129, Department of Psychiatry, Virginia Commonwealth University, Richmond, VA, US

130, Health Care Policy, Harvard Medical School, Boston, MA, US

131, Department of Psychiatry, University of Toronto, Toronto, ON, CA

132, Department of Pharmacology & Toxicology, University of Toronto, Toronto, ON, CA

133, Department of Genetics, Rutgers University, Piscataway, NJ, US

134, Department of Psychiatry, Perelman School of Medicine, University of Pennsylvania, Philadelphia, PA, US

135, Mental Illness Research, Education and Clinical Center, Crescenz VA Medical Center, Philadelphia, PA, US

136, Estonian Genome Centre, Institute of Genomics, University of Tartu, Tartu, EE

137, Department of Women's and Children's Health, Uppsala University, Uppsala, SE

138, Department of Epidemiology and Health Systems, Center for Primary Care and Public Health, Lausanne, VD, CH

139, Swiss Institute of Bioinformatics, Lausanne, VD, CH

140, Department of Computational Biology, University of Lausanne, Lausanne, VD, CH

141, Institute for Molecular Medicine Finland - FIMM, University of Helsinki, Helsinki, FI

142, Department of Psychiatry, Washington University School of Medicine in St. Louis, St. Louis, MO, US

143, Psychiatry, Veterans Affairs Connecticut Healthcare System, West Haven, CT, US

144, Department of Psychiatry, Yale University, New Haven, CT, US

145, Department of Epidemiology and Biostatistics, School of Public Health, Peking University, Beijing, CN

146, Peking University Center for Public Health and Epidemic Preparedness & Response, Peking University, Beijing, CN

147, Mental Health, NHS 24, Glasgow, UK

148, Royal Edinburgh Hospital, NHS Lothian, Edinburgh, UK

149, Department of Psychiatry and Psychotherapy, University of Bonn, Bonn, DE

150, Center for Translational and Computational Neuroimmunology, Columbia University Medical Center, New York, NY, US

151, Institute for Translational Neuroscience, University of Münster, Münster, NRW, DE

152, Novo Nordisk Foundation Center for Basic Metabolic Research, Faculty of Health Science, Copenhagen University, Copenhagen, DK

153, Department of Nursing, Faculty of Health Sciences and Institute of Neurosciences, Biomedical Research Centre (CIBM), University of Granada, Granada, ES

154, School of Public Health, University of Queensland, Brisbane, QLD, AU

155, DZHK (German Centre for Cardiovascular Research), Partner Site Greifswald, Greifswald, MV, DE

156, Institute of Clinical Chemistry and Laboratory Medicine, University Medicine Greifswald, Greifswald, MV, DE

157, Department of Psychiatry, University of Marburg, Marburg, DE

158, Department of Clinical Immunology, Aalborg University Hospital, Aalborg, DK

159, Department of Epidemiology, University of Groningen, University Medical Center Groningen, Groningen, NL

160, Department of Health, Science and Technology, Aalborg University, Aalborg, DK

161, Centre for Integrative Sequencing, iSEQ, Aarhus University, Aarhus, DK

162, Department of Biomedicine-Human Genetics, Aarhus University, Aarhus, DK

163, Population Health, QIMR Berghofer Medical Research Institute, Brisbane, QLD, AU

164, The Fraser Institute, Faculty of Medicine, University of Queensland, Brisbane, QLD, AU

165, Humus, Reykjavik, IS

166, Department of Clinical Medicine, University of Copenhagen, Copenhagen, CPH, DK

167, Department of Population Health Sciences, University of Leicester, Leicester, UK

168, Department of Psychiatry, Universidade Federal de Sao Paulo, Sao Paulo, SP, BR

169, Department of Psychiatry, Rutgers University, Piscataway, NJ, US

170, Department of Clinical Immunology, Zealand University Hospital, Køge, DK

171, Department of Psychiatry and Behavioral Sciences, SUNY Downstate Health Sciences University, Brooklyn, NY, US

172, NMD Pharma, Lexington, MA, US

173, Psychiatry, University of Pittsburgh Medical Centre, Pittsburgh, PA, US

174, Psychiatry, Universidade Federal do Rio Grande do Sul, Porto Alegre, BR

175, Department of Psychiatry, University Medical Center Groningen, Groningen, NL

176, Research School of Behavioural and Cognitive Neurosciences (BCN), University of Groningen, Groningen, NL

177, Institute of Biological Psychiatry, Mental Health Center Sct. Hans, Mental Health Services Capital Region of Denmark, Copenhagen, DK

178, Neurogenomics Division, The Translational Genomics Research Institute (TGEN), Phoenix, AZ, US

179, Human Genetics Branch, NIMH Division of Intramural Research Programs, Bethesda, MD, US

180, Department of Psychiatry and Psychotherapy, University Medical Center Göttingen, Goettingen, NI, DE

181, Division of Cancer Epidemiology and Genetics, National Cancer Institute, Bethesda, MD, US

182, School of Medicine and Dentistry, James Cook University, Townsville, QLD, AU

183, Division of Mental Health and Addiction, Oslo University Hospital, Oslo, OSL, NO

184, NORMENT, Institute of Clinical Medicine, University of Oslo, Oslo, OSL, NO

185, Institute of Health and Wellbeing, University of Glasgow, Glasgow, UK

186, Division of Population Health and Genomics, Ninewells Hospital and School of Medicine, University of Dundee, Dundee, UK

187, Faculty of Medicine, University of Iceland, Reykjavik, IS

188, SAMRC Unit on Risk & Resilience in Mental Disorders, Department of Psychiatry and Mental Health, University of Cape Town, Cape Town, SA

189, Department of Psychiatry and Psychotherapy, University of Marburg, Marburg, HE, DE

190, Department of Psychiatry, Psychotherapy and Psychosomatics, Dr. Fontheim Mentale Gesundheit, Liebenburg, DE

191, Institute of Epidemiology and Social Medicine, University of Münster, Münster, NRW, DE

192, Institute for Community Medicine, University Medicine Greifswald, Greifswald, MV, DE

193, Wolfson Centre for Young People's Mental Health, Division of Psychological Medicine and Clinical Neurosciences, Cardiff University, Cardiff, WLS, UK

194, Department of Biological Sciences, Purdue University, West Lafayette, IN, US

195, Imperial College BHF Centre for Research Excellence, Imperial College London, London, UK

196, Genomics Program, University of South Florida College of Public Health, Tampa, FL, US

197, Department of Internal Medicine, Erasmus University Medical Center Rotterdam, Rotterdam, NL

198, Management Section, Statens Serum Institut, Copenhagen, DK

199, Department of Clinical Medicine, University of Copenhagen, Copenhagen, DK

200, Xperimed LLC, Basel, CH

201, Psychiatry, USUHS, Bethesda, US

202, Department of Psychiatry and Psychotherapy, University Medicine Greifswald, Greifswald, MV, DE

203, Department of Psychiatry, Leiden University Medical Center, Leiden, NL

204, Department of Epidemiology and Population Health, Albert Einstein College of Medicine, Bronx, NY, US

205, Department of Psychiatry, Columbia University College of Physicians and Surgeons, New York, NY, US

206, Division of Epidemiology, New York State Psychiatric Institute, New York, NY, US

207, Department of Neurology, Oslo University Hospital, Oslo, NO

208, HUNT All-In Psychiatry

209, China Kadoorie Biobank Collaborative Group

210, Genes & Health Research Team

211, KG Jebsen Centre for Neurodevelopmental Research, University of Oslo, Oslo, OSL, NO

212, Department of Psychiatry, University of Melbourne, Melbourne, VIC, AU

213, Florey Institute of Neuroscience and Mental Health, University of Melbourne, Melbourne, VIC, AU

214, Department of Psychiatry, University of Münster, Münster, NRW, DE

215, Computational Health Centre, Helmholtz Zentrum München, Neuherberg, DE

216, School of Medicine, Technical University of Munich, Munich, BY, DE

217, Helmholtz Pioneer Campus, Helmholtz Zentrum München, Neuherberg, DE

218, Department of Psychiatry, University of Vermont, Burlington, VT, US

219, Department of Cellular, Computational and Integrative Biology, Università degli Studi di Trento, Trento, IT

220, Imperial College Biomedical Research Centre, Imperial College London, London, UK

221, Department of Psychiatry, Psychosomatics and Psychotherapy, Julius-Maximilians-Universität Würzburg, Würzburg, DE

222, Department of Genetics, Department of Neuroscience, Yale University School of Medicine, New Haven, CT, US

223, Psychiatry, Kaiser Permanente Northern California, San Francisco, CA, US

224, K. G. Jebsen Center for Genetic Epidemiology, Department of Public Health and Nursing, Faculty of Medicine and Health Sciences, Norwegian University of Science and Technology (NTNU), Trondheim, NO

225, HUNT Research Center, Department of Public Health and Nursing, Faculty of Medicine and Health Sciences, Norwegian University of Science and Technology (NTNU), Trondheim, NO

226, Department of Research, Innovation and Education, St. Olavs Hospital, Trondheim University Hospital, Trondheim, NO

227, NIHR Leicester Biomedical Research Centre, Glenfield Hospital, Leicester, UK

228, Pathophysiology of Psychiatric Diseases, INSERM, Univ Paris Cité, GHU Paris, Paris, FR

229, Department of Psychiatry & Behavioral Sciences, Stanford University, Stanford, CA, US

230, Neuroscience Therapeutic Area, Janssen Research and Development, LLC, Titusville, NJ, US

231, Second Opinion Outpatient Clinic, GGNet Mental Health, Warnsveld, NL

232, Child and Youth Mental Health Service, Children's Health Queensland Hospital and Health Service, Brisbane, QLD, AU

233, Psychosis Research Unit, Aarhus University Hospital-Psychiatry, Aarhus, DK

234, Department of Psychiatry, Psychosomatics and Psychotherapy, University Hospital of Würzburg, Würzburg, DE

235, Munich Cluster for Systems Neurology (SyNergy), Munich, BY, DE

236, University of Liverpool, Liverpool, UK

237, Human Genetics and Computational Biomedicine, Pfizer Global Research and Development, Groton, CT, US

238, Centre for Quantitative Health, Massachusetts General Hospital, Boston, MA, US

239, Child and Adolescent Psychiatry, Amsterdam UMC, Vrije Universiteit Amsterdam, Amsterdam, NL

240, Complex Trait Genetics, Vrije Universiteit Amsterdam, Amsterdam, NL

241, Wolfson Centre for Young People's Mental Health, Division of Psychological Medicine and Clinical Neurosciences, Cardiff University, Cardiff, UK

242, Department of Biochemistry and Molecular Biology II, Faculty of Pharmacy and Institute of Neurosciences, Biomedical Research Centre (CIBM), University of Granada, Granada, ES

243, Department of Clinical Neuroscience, Karolinska Institutet, Stockholm, SE

244, Division of Research, Kaiser Permanente Northern California, Oakland, CA, US

245, Department of Psychiatry, University of Michigan, Ann Arbor, MI, US

246, Stanley Center for Psychiatric Research, Broad Institute of MIT and Harvard, Cambridge, MA, US

247, Psychiatric and Neurodevelopmental Genetics Unit, Massachusetts General Hospital, Boston, MA, US

248, Psychiatry, UCSD School of Medicine, La Jolla, CA, US

249, Public Health, UCSD School of Public Health, La Jolla, CA, US

250, Psychiatry, Veterans Affairs San Diego Healthcare System, San Diego, CA, US

251, Departments of Genetics and Psychiatry, University of North Carolina at Chapel Hill, Chapel Hill, NC, US

252, Child and Adolescent Psychiatry, Erasmus University Medical Center Rotterdam, Rotterdam, NL

253, Social and Behavioral Science, Harvard T.H. Chan School of Public Health, Boston, MA, US

254, Psychiatry, Dalhousie University, Halifax, NS, CA

255, Institute of Biological Psychiatry, Mental Health Center Sct. Hans, Copenhagen University Hospital, Mental Health Services, Copenhagen, DK

256, GLOBE Institute, Lundbeck Foundation Centre for Geogenetics, University of Copenhagen, Copenhagen, DK

257, Queensland Brain Institute, University of Queensland, Brisbane, QLD, AU

258, Department of Medical & Molecular Genetics, King's College London, London, UK

259, Institute for Genomics and Cancer, University of Edinburgh, Edinburgh, UK

## References

Als, T. D., Kurki, M. I., Grove, J., Voloudakis, G., Therrien, K., Tasanko, E., Nielsen, T. T., Naamanka, J., Veerapen, K., Levey, D. F., Bendl, J., Bybjerg-Grauholm, J., Zeng, B., Demontis, D., Rosengren, A., Athanasiadis, G., Bækved-Hansen, M., Qvist, P., Bragi Walters, G., … Børglum, A. D. (2023). Depression pathophysiology, risk prediction of recurrence and comorbid psychiatric disorders using genome-wide analyses. *Nature Medicine*, *29*(7), 1832–1844. https://doi.org/10.1038/s41591-023-02352-1

Boyd, A., Golding, J., Macleod, J., Lawlor, D. A., Fraser, A., Henderson, J., Molloy, L., Ness, A., Ring, S., & Davey Smith, G. (2013). Cohort Profile: The ‘Children of the 90s’—the index offspring of the Avon Longitudinal Study of Parents and Children. *International Journal of Epidemiology*, *42*(1), 111–127. https://doi.org/10.1093/ije/dys064

Bycroft, C., Freeman, C., Petkova, D., Band, G., Elliott, L. T., Sharp, K., Motyer, A., Vukcevic, D., Delaneau, O., O’Connell, J., Cortes, A., Welsh, S., Young, A., Effingham, M., McVean, G., Leslie, S., Allen, N., Donnelly, P., & Marchini, J. (2018). The UK Biobank resource with deep phenotyping and genomic data. *Nature*, *562*(7726), 203–209. https://doi.org/10.1038/s41586-018-0579-z

Byrne, E. M., Kirk, K. M., Medland, S. E., McGrath, J. J., Colodro-Conde, L., Parker, R., Cross, S., Sullivan, L., Statham, D. J., Levinson, D. F., Licinio, J., Wray, N. R., Hickie, I. B., & Martin, N. G. (2020). Cohort profile: The Australian genetics of depression study. *BMJ Open*, *10*(5), e032580. https://doi.org/10.1136/bmjopen-2019-032580

Chang, C. C., Chow, C. C., Tellier, L. C. A. M., Vattikuti, S., Purcell, S. M., & Lee, J. J. (2015). Second-generation PLINK: rising to the challenge of larger and richer datasets. *GigaScience*, *4*(1), 7. https://doi.org/10.1186/s13742-015-0047-8

Dashti, H. S., Redline, S., & Saxena, R. (2018). Polygenic risk score identifies associations between sleep duration and diseases determined from an electronic medical record biobank. *Sleep*, *42*, 1–10. https://doi.org/10.1093/sleep/zsy247

Davis, K. A. S., Coleman, J. R. I., Adams, M., Allen, N., Breen, G., Cullen, B., Dickens, C., Fox, E., Graham, N., Holliday, J., Howard, L. M., John, A., Lee, W., McCabe, R., McIntosh, A., Pearsall, R., Smith, D. J., Sudlow, C., Ward, J., … Hotopf, M. (2020). Mental health in UK Biobank – development, implementation and results from an online questionnaire completed by 157 366 participants: A reanalysis. *BJPsych Open*, *6*(2), e18. https://doi.org/10.1192/bjo.2019.100

Fernandez-Pujals, A. M., Adams, M. J., Thomson, P., McKechanie, A. G., Blackwood, D. H. R., Smith, B. H., Dominiczak, A. F., Morris, A. D., Matthews, K., Campbell, A., Linksted, P., Haley, C. S., Deary, I. J., Porteous, D. J., MacIntyre, D. J., & McIntosh, A. M. (2015). Epidemiology and Heritability of Major Depressive Disorder, Stratified by Age of Onset, Sex, and Illness Course in Generation Scotland: Scottish Family Health Study (GS:SFHS). *PLoS ONE*, *10*(11), e0142197. https://doi.org/10.1371/journal.pone.0142197

Fraser, A., Macdonald-Wallis, C., Tilling, K., Boyd, A., Golding, J., Davey Smith, G., Henderson, J., Macleod, J., Molloy, L., Ness, A., Ring, S., Nelson, S. M., & Lawlor, D. A. (2013). Cohort Profile: The Avon Longitudinal Study of Parents and Children: ALSPAC mothers cohort. *International Journal of Epidemiology*, *42*(1), 97–110. https://doi.org/10.1093/ije/dys066

Grotzinger, A. D., Fuente, J. de la, Privé, F., Nivard, M. G., & Tucker-Drob, E. M. (2022). Pervasive Downward Bias in Estimates of Liability-Scale Heritability in Genome-wide Association Study Meta-analysis: A Simple Solution. *Biological Psychiatry*, S0006322322013166. https://doi.org/10.1016/j.biopsych.2022.05.029

Grotzinger, A. D., Mallard, T. T., Akingbuwa, W. A., Ip, H. F., Adams, M. J., Lewis, C. M., McIntosh, A. M., Grove, J., Dalsgaard, S., Lesch, K.-P., Strom, N., Meier, S. M., Mattheisen, M., Børglum, A. D., Mors, O., Breen, G., iPSYCH, Mattheisen, M., Mors, O., … Nivard, M. G. (2022). Genetic architecture of 11 major psychiatric disorders at biobehavioral, functional genomic and molecular genetic levels of analysis. *Nature Genetics*, *54*(5), 548–559. https://doi.org/10.1038/s41588-022-01057-4

Hickie, I. B., Davenport, T. A., Hadzi‐Paviovic, D., Koschera, A., Naismith, S. L., Scott, E. M., & Wilhelm, K. A. (2001). Development of a simple screening tool for common mental disorders in general practice. *Medical Journal of Australia*, *175*(S1). https://doi.org/10.5694/j.1326-5377.2001.tb143784.x

Johnston, K. J. A., Adams, M. J., Nicholl, B. I., Ward, J., Strawbridge, R. J., Ferguson, A., McIntosh, A. M., Bailey, M. E. S., & Smith, D. J. (2019). Genome-wide association study of multisite chronic pain in UK Biobank. *PLOS Genetics*, *15*(6), e1008164. https://doi.org/10.1371/journal.pgen.1008164

Lam, M., Awasthi, S., Watson, H. J., Goldstein, J., Panagiotaropoulou, G., Trubetskoy, V., Karlsson, R., Frei, O., Fan, C.-C., De Witte, W., Mota, N. R., Mullins, N., Brügger, K., Lee, S. H., Wray, N. R., Skarabis, N., Huang, H., Neale, B., Daly, M. J., … Ripke, S. (2020). RICOPILI: Rapid Imputation for COnsortias PIpeLIne. *Bioinformatics (Oxford, England)*, *36*(3), 930–933. https://doi.org/10.1093/bioinformatics/btz633

Leitsalu, L., Haller, T., Esko, T., Tammesoo, M.-L., Alavere, H., Snieder, H., Perola, M., Ng, P. C., Mägi, R., Milani, L., Fischer, K., & Metspalu, A. (2015). Cohort Profile: Estonian Biobank of the Estonian Genome Center, University of Tartu. *International Journal of Epidemiology*, *44*(4), 1137–1147. https://doi.org/10.1093/ije/dyt268

Lewis, G., Pelosi, A. J., Araya, R., & Dunn, G. (1992). Measuring psychiatric disorder in the community: A standardized assessment for use by lay interviewers. *Psychological Medicine*, *22*(2), 465–486. https://doi.org/10.1017/S0033291700030415

Liu, M., Jiang, Y., Wedow, R., Li, Y., Brazel, D. M., Chen, F., Datta, G., Davila-Velderrain, J., McGuire, D., Tian, C., Zhan, X., Agee, M., Alipanahi, B., Auton, A., Bell, R. K., Bryc, K., Elson, S. L., Fontanillas, P., Furlotte, N. A., … HUNT All-In Psychiatry. (2019). Association studies of up to 1.2 million individuals yield new insights into the genetic etiology of tobacco and alcohol use. *Nature Genetics*, *51*(2), 237–244. https://doi.org/10.1038/s41588-018-0307-5

Major Depressive Disorder Working Group of the Psychiatric GWAS Consortium. (2013). A mega-analysis of genome-wide association studies for major depressive disorder. *Molecular Psychiatry*, *18*, 497–511. https://doi.org/10.1038/mp.2012.21

Mitchell, B. L., Campos, A. I., Whiteman, D. C., Olsen, C. M., Gordon, S. D., Walker, A. J., Dean, O. M., Berk, M., Hickie, I. B., Medland, S. E., Wray, N. R., Martin, N. G., & Byrne, E. M. (2022). The Australian Genetics of Depression Study: New Risk Loci and Dissecting Heterogeneity Between Subtypes. *Biological Psychiatry*, *92*(3), 227–235. https://doi.org/10.1016/j.biopsych.2021.10.021

Mullins, N., Forstner, A. J., O’Connell, K. S., Coombes, B., Coleman, J. R. I., Qiao, Z., Als, T. D., Bigdeli, T. B., Børte, S., Bryois, J., Charney, A. W., Drange, O. K., Gandal, M. J., Hagenaars, S. P., Ikeda, M., Kamitaki, N., Kim, M., Krebs, K., Panagiotaropoulou, G., … HUNT All-In Psychiatry. (2021). Genome-wide association study of more than 40,000 bipolar disorder cases provides new insights into the underlying biology. *Nature Genetics*, *53*(6), 817–829. https://doi.org/10.1038/s41588-021-00857-4

Nagel, M., Jansen, P. R., Stringer, S., Watanabe, K., de Leeuw, C. A., Bryois, J., Savage, J. E., Hammerschlag, A. R., Skene, N. G., Muñoz-Manchado, A. B., Agee, M., Alipanahi, B., Auton, A., Bell, R. K., Bryc, K., Elson, S. L., Fontanillas, P., Furlotte, N. A., Hinds, D. A., … andMe Research, T. (2018). Meta-analysis of genome-wide association studies for neuroticism in 449,484 individuals identifies novel genetic loci and pathways. *Nature Genetics*, *50*(7), 920–927. https://doi.org/10.1038/s41588-018-0151-7

Nievergelt, C. M., Maihofer, A. X., Klengel, T., Atkinson, E. G., Chen, C.-Y., Choi, K. W., Coleman, J. R. I., Dalvie, S., Duncan, L. E., Gelernter, J., Levey, D. F., Logue, M. W., Polimanti, R., Provost, A. C., Ratanatharathorn, A., Stein, M. B., Torres, K., Aiello, A. E., Almli, L. M., … Koenen, K. C. (2019). International meta-analysis of PTSD genome-wide association studies identifies sex- and ancestry-specific genetic risk loci. *Nature Communications*, *10*(1), 4558. https://doi.org/10.1038/s41467-019-12576-w

Okbay, A., Wu, Y., Wang, N., Jayashankar, H., Bennett, M., Nehzati, S. M., Sidorenko, J., Kweon, H., Goldman, G., Gjorgjieva, T., Jiang, Y., Hicks, B., Tian, C., Hinds, D. A., Ahlskog, R., Magnusson, P. K. E., Oskarsson, S., Hayward, C., Campbell, A., … Young, A. I. (2022). Polygenic prediction of educational attainment within and between families from genome-wide association analyses in 3 million individuals. *Nature Genetics*, *54*(4), 437–449. https://doi.org/10.1038/s41588-022-01016-z

Pulit, S. L., Stoneman, C., Morris, A. P., Wood, A. R., Glastonbury, C. A., Tyrrell, J., Yengo, L., Ferreira, T., Marouli, E., Ji, Y., Yang, J., Jones, S., Beaumont, R., Croteau-Chonka, D. C., Winkler, T. W., GIANT Consortium, Hattersley, A. T., Loos, R. J. F., Hirschhorn, J. N., … Lindgren, C. M. (2019). Meta-analysis of genome-wide association studies for body fat distribution in 694 649 individuals of European ancestry. *Human Molecular Genetics*, *28*(1), 166–174. https://doi.org/10.1093/hmg/ddy327

Smith, B. H., Campbell, A., Linksted, P., Fitzpatrick, B., Jackson, C., Kerr, S. M., Deary, I. J., MacIntyre, D. J., Campbell, H., & McGilchrist, M. (2012). Cohort profile: Generation Scotland: Scottish Family Health Study (GS: SFHS). The study, its participants and their potential for genetic research on health and illness. *Int J Epidemiol*, *42*(2), 689–700.

Sudlow, C., Gallacher, J., Allen, N., Beral, V., Burton, P., Danesh, J., Downey, P., Elliott, P., Green, J., Landray, M., Liu, B., Matthews, P., Ong, G., Pell, J., Silman, A., Young, A., Sprosen, T., Peakman, T., & Collins, R. (2015). UK Biobank: An Open Access Resource for Identifying the Causes of a Wide Range of Complex Diseases of Middle and Old Age. *PLOS Med*, *12*(3), e1001779. https://doi.org/10.1371/journal.pmed.1001779

The 1000 Genomes Project Consortium. (2015). A global reference for human genetic variation. *Nature*, *526*, 68–74. https://doi.org/10.1038/nature15393

Walters, R. K., Adams, M. J., Adkins, A. E., Aliev, F., Bacanu, S.-A., Batzler, A., Bertelsen, S., Biernacka, J., Bigdeli, T. B., Chen, L.-S., Clarke, T.-K., Chou, Y.-L., Degenhardt, F., Docherty, A. R., Fontanillas, P., Foo, J., Fox, L., Frank, J., Giegling, I., … Agrawal, A. (2018). Trans-ancestral GWAS of alcohol dependence reveals common genetic underpinnings with psychiatric disorders. *BioRxiv*, 257311. https://doi.org/10.1101/257311

Wray, N. R., Ripke, S., Mattheisen, M., Trzaskowski, M., Byrne, E. M., Abdellaoui, A., Adams, M. J., Agerbo, E., Air, T. M., Andlauer, T. M. F., Bacanu, S.-A., Bækvad-Hansen, M., Beekman, A. F. T., Bigdeli, T. B., Binder, E. B., Blackwood, D. R. H., Bryois, J., Buttenschøn, H. N., Bybjerg-Grauholm, J., … Sullivan, P. F. (2018). Genome-wide association analyses identify 44 risk variants and refine the genetic architecture of major depression. *Nature Genetics*, *50*(5), 668–681. https://doi.org/10.1038/s41588-018-0090-3

Zhou, W., Nielsen, J. B., Fritsche, L. G., Dey, R., Gabrielsen, M. E., Wolford, B. N., LeFaive, J., VandeHaar, P., Gagliano, S. A., Gifford, A., Bastarache, L. A., Wei, W.-Q., Denny, J. C., Lin, M., Hveem, K., Kang, H. M., Abecasis, G. R., Willer, C. J., & Lee, S. (2018). Efficiently controlling for case-control imbalance and sample relatedness in large-scale genetic association studies. *Nature Genetics*, *50*(9), 1335–1341. https://doi.org/10.1038/s41588-018-0184-y
